# Nonautonomous Young Differential Equations Revisited

**DOI:** 10.1007/s10884-017-9634-y

**Published:** 2017-12-20

**Authors:** Nguyen Dinh Cong, Luu Hoang Duc, Phan Thanh Hong

**Affiliations:** 1 Institute of Mathematics, Vietnam Academy of Science and Technology, Hanoi, Vietnam; 2 Max-Planck-Institut für Mathematik in den Naturwissenschaften, Leipzig, Germany; 3 Thang Long University, Hanoi, Vietnam

**Keywords:** Stochastic differential equations (SDE), Fractional Brownian motion (fBm), Young integral, *p*-variation

## Abstract

In this paper we prove that under mild conditions a nonautonomous Young differential equation possesses a unique solution which depends continuously on initial conditions. The proofs use estimates in *p*-variation norms, the construction of greedy sequence of times, and Gronwall-type lemma with the help of Shauder theorem of fixed points.

## Introduction

This paper deals with the Young differential equation of the form

1.1 $$\begin{aligned} dx_t = f(t,x_t)dt+g(t,x_t)d\omega _t, \ t\ge 0 \end{aligned}$$

where $f: \mathbb {R}\times \mathbb {R}^d \rightarrow \mathbb {R}^d$ and $g: \mathbb {R}\times \mathbb {R}^d \rightarrow \mathbb {R}^{d \times m}$ are continuous functions, $\omega$ is a $\mathbb {R}^m$-valued function of finite *p*-variation norm for some $1<p<2$. Such type of system is generated from stochastic differential equations driven by fractional Brownian noises, as seen e.g. in [20]. Equation (1.1) is understood in the integral form

1.2 $$\begin{aligned} x_t=x_{0} +\int _0^t f(s,x_s) ds + \int _0^tg(s,x_s)d\omega _s,\ t\ge 0, \end{aligned}$$

where the first integral is of Riemannian type, meanwhile the second integral can be defined in the Young sense [23]. The existence and uniqueness of the solution of (1.2) are studied by several authors. When *f*, *g* are time-independent, system (1.2) is proved in [23] and [22] and [18] to have a unique solution in a certain space of continuous functions with bounded *p*-variation.

The result is then generalized for the case $2\le p<3$ in Lyons’ seminal paper [19] in which rough path theory is introduced to define the second integral in (1.2) and also the integration equation (see [8, 16] and [17]). An alternative theory of controlled paths in Gubinelli’s work [10] simplifies and generalizes the concept of integration and differential equations, leading to the concept of rough differential equations (see recent works in [3] and [2] for $2\le p<3$, or [14] for controlled differential equations as Young integrals). According to their settings, *f*, *g* are time independent and *g* is often assumed to be differentiable upto a certain order and bounded in its derivatives.

A different approach following Zähle [24] by using fractional derivatives can be seen in [21] which derives very weak conditions for time varying *f* and *g* in (1.1), in particular *g* need to be only $C^1$ with bounded and Hölder continuous first derivative, to ensure the existence and uniqueness of the solution in the space of Hölder continuous functions. Later, one finds that there is a connection between the rough path approach and the techniques in fractional calculus, see e.g. [11] and [12].

Our aim in this paper is to close the gap between the two methods for nonautonomous Young equations by proving that, under similar assumptions to those of Nualart and Rascanu [21], the existence and uniqueness theorem for system (1.1) still holds in the space of continuous functions with bounded *p*-variation norm. For that to work, we construct the so-called *greedy* sequence of times (see [4, Definition 4.7]) such that the solution can be proved to exists uniquely in each interval of the consecutive times of the greedy sequence, and is then concatenated to form a global solution. It is important to note that since we are using estimates for *p*-variation norms, we do not apply the classical arguments of contraction mappings, but use Shauder-Tychonoff fixed point theorem as seen in [18] and a Gronwall-type lemma.

Another issue is the generation of flow which was asserted in [17] for the autonomous systems. The idea is to construct the shift dynamical system in the extended space of finite *p*-variation norm for the whole real line time, and to prove that the system generates a nonautonomous dynamical system satisfying the cocycle property (see [3]). When applying to stochastic differential equations driven by fractional Brownian motions, by considering an appropriate probability space, one can prove that the system generates a random dynamical system (see [3, 5, 9]). However in the nonautonomous situation, one only expects a generation of a two-parameter flow on the phase space.

The paper is organized as follows. In Sect. 2, the Young integral is introduced and a version of greedy sequence of times is presented. In Sect. 3, we prove the existence and uniqueness of the global solution of system (1.2) in Theorem 3.6, for this we need to formulate a Gronwall-type lemma. Proposition 3.7 gives an estimate of *q*-var norm of solution via *p*-var norm of the driver $\omega$. We also prove the existence and uniqueness of the solution of the backward equation (3.23) in Theorem 3.8. In Sect. 4, the fact in Theorem 4.1 that two trajectories do not intersect helps to conclude that the Cauchy operator or the Ito map of (1.2) generates a continuous two parameter flow. In the autonomous case it generates a continuous nonautonomous dynamical system which helps to form a topological skew product flow.

## Preliminaries

### Young Integral

In this section we recall some facts about Young integral, more details can be seen in [8]. Let $C([a,b],\mathbb {R}^d)$ denote the space of all continuous paths $x:\;[a,b] \rightarrow \mathbb {R}^d$ equipped with sup norm $\Vert \cdot \Vert _{\infty ,[a,b]}$ given by $\Vert x\Vert _{\infty ,[a,b]}=\sup _{t\in [a,b]} |x_t|$, where $|\cdot |$ is the Euclidean norm in $\mathbb {R}^d$. For $p\ge 1$ and $[a,b] \subset \mathbb {R}$, a continuous path $x:[a,b] \rightarrow \mathbb {R}^d$ is of finite *p*-variation if

2.1 $$\begin{aligned} \left| \left| \left| x\right| \right| \right| _{p\text {-var},[a,b]} :=\left( \sup _{\Pi (a,b)}\sum _{i=1}^n|x_{t_{i+1}}-x_{t_i}|^p\right) ^{1/p} < \infty , \end{aligned}$$

where the supremum is taken over the whole class of finite partition of [*a*, *b*]. The subspace $\widehat{C}^p([a,b],\mathbb {R}^d)\subset C([a,b],\mathbb {R}^d)$ of all paths *x* with finite *p*-variation and equipped with the *p*-var norm

$$\begin{aligned} \Vert x\Vert _{p\text {-var},[a,b]}:= |x_a|+\left| \left| \left| x\right| \right| \right| _{p\text {-var},[a,b]}, \end{aligned}$$

is a nonseparable Banach space [8, Theorem 5.25, p. 92]. Notice that if $x\in \widehat{C}^p([a,b],\mathbb {R}^d)$ then the mapping $(s,t)\rightarrow \left| \left| \left| x\right| \right| \right| _{p\text {-var},[s,t]}$ is continuous on the simplex $\Delta [a,b]:=\{(s,t)|a\le s\le t\le b\}$, see [8, Proposition 5.8, p. 80].

Furthermore, the closure of $C^\infty ([a,b],\mathbb {R}^d)$ in $\widehat{C}^p([a,b],\mathbb {R}^d)$ is a separable Banach space denoted by $\widehat{C}^{0,p}([a,b],\mathbb {R}^d)$ which can be defined as the space of all continuous paths *x* such that

$$\begin{aligned} \lim \limits _{\delta \rightarrow 0} \sup _{\Pi (a,b), |\Pi | \le \delta } \sum _i |x_{t_{i+1}}-x_{t_i}|^p =0. \end{aligned}$$

It is easy to prove (see [8, Corollary 5.33, p. 98]) that for $1\le p< p'$ we have

$$\begin{aligned} \widehat{C}^p([a,b],\mathbb {R}^d)\subset \widehat{C}^{0,p'}([a,b],\mathbb {R}^d). \end{aligned}$$

Also, for $0<\alpha \le 1$ denote by $C^{\alpha \text {-Hol}}([a,b],\mathbb {R}^d)$ the Banach space of all Hölder continuous paths $x:[a,b]\rightarrow \mathbb {R}^d$ with exponential $\alpha$, equipped with the norm

2.2 $$\begin{aligned} \Vert x\Vert _{\alpha \text {-Hol},[a,b]}:= & {} |x_a| + \left| \left| \left| x\right| \right| \right| _{\alpha \text {-Hol},[a,b]}\nonumber \\= & {} |x_a| +\sup _{(s,t)\in \Delta [a,b]} \frac{|x_t-x_s|}{(t-s)^{\alpha }}< \infty . \end{aligned}$$

Clearly, if $x\in C^{\alpha \text {-Hol}}([a,b],\mathbb {R}^d)$ then for all $s,t\in [a,b]$ we have

$$\begin{aligned} |x_t-x_s|\le \left| \left| \left| x\right| \right| \right| _{\alpha \text {-Hol},[a,b]} |t-s|^\alpha . \end{aligned}$$

Hence, for all *p* such that $p\alpha \ge 1$ we have

2.3 $$\begin{aligned} \left| \left| \left| x\right| \right| \right| _{p\text {-var},[a,b]}\le \left| \left| \left| x\right| \right| \right| _{\alpha \text {-Hol},[a,b]} (b-a)^\alpha <\infty . \end{aligned}$$

Therefore, $$C^{1/p\text {-Hol}}([a,b],\mathbb {R}^d)\subset \widehat{C}^p([a,b],\mathbb {R}^d)$$.

As introduced in [21], the Besov space $W_b^{1/p,\infty }([a,b],\mathbb {R}^d)$ of measurable functions $g :[a,b] \rightarrow \mathbb {R}^d$ such that

$$\begin{aligned} \sup _{a<s<t<b}\left( \frac{|g_t-g_s|}{(t-s)^{1/p}}+\int _s^t\frac{|g_y-g_s|}{(y-s)^{1+1/p}}dy\right) <\infty \end{aligned}$$

is a subspace of $C^{1/p\text {-Hol}}([a,b],\mathbb {R}^d)$. Hence $W_b^{1/p,\infty }([a,b],\mathbb {R}^d)\subset \widehat{C}^p([a,b],\mathbb {R}^d)$.

#### Lemma 2.1

Let $x\in \widehat{C}^{p}([a,b],\mathbb {R}^d)$, $p\ge 1$. If $a = a_1<a_2<\cdots < a_k = b$, then

$$\begin{aligned} \sum _{i=1}^{k-1}\left| \left| \left| x\right| \right| \right| ^p_{p\text {-}\mathrm{var},[a_i,a_{i+1}]}\le \left| \left| \left| x\right| \right| \right| ^p_{p\text {-}\mathrm{var},[a_1,a_k]}\le (k-1)^{p-1}\sum _{i=1}^{k-1}\left| \left| \left| x\right| \right| \right| ^p_{p\text {-}\mathrm{var},[a_i,a_{i+1}]}. \end{aligned}$$

#### Proof

The proof is similar to the one in [8, p. 84], by using triangle inequality and power means inequality

$$\begin{aligned} \frac{1}{n}\sum _{i=1}^n z_i \le \left( \frac{1}{n}\sum _{i=1}^nz_i^r\right) ^{1/r},\quad \forall z_i\ge 0, r\ge 1. \end{aligned}$$

$$\square $$

#### Definition 2.2

A continuous map $\overline{\omega }: \Delta [a,b]\longrightarrow \mathbb {R}^+$ is called a control if it is zero on the diagonal and superadditive, i.e

(i), For all $$t\in [a,b]$$, $$\overline{\omega }_{t,t}=0$$,
(ii), For all $s\le t\le u$ in [*a*, *b*], $\overline{\omega }_{s,t}+\overline{\omega }_{t,u}\le \overline{\omega }_{s,u}$.

The functions $(s,t)\longrightarrow (t-s)^{\theta }$ with $\theta \ge 1$, and $(s,t)\longrightarrow \left| \left| \left| x\right| \right| \right| ^q_{p\text {-var},[s,t]}$, where *x* is of bounded *p*-variation norm on [*a*, *b*] and $q\ge p$ are some examples of control function. The following lemma gives a useful property of controls in relation with variations of a path (see [8] for more properties of control functions).

#### Lemma 2.3

Let $\overline{\omega }^j$ be a finite sequence of control functions on [0, *T*], $C_j>0,\;\;j=\overline{1,k}$, $p\ge 1$ and $x:[0,T]\rightarrow \mathbb {R}^d$ be a continuous path satisfying $|x_t-x_s|\le \sum _{i=j}^k C_j \overline{\omega }^j(s,t)^{1/p},\;\; \forall s<t \in [0,T]$, then

2.4 $$\begin{aligned} \left| \left| \left| x\right| \right| \right| _{p\text {-}\mathrm{{var}},[s,t]}\le \sum _{j=1}^k C_j \overline{\omega }^j(s,t)^{1/p},\;\; \forall s<t \in [0,T]. \end{aligned}$$

#### Proof

Consider an arbitrary finite partition $\Pi = (s_i)$, $i=0\ldots , n+1$, of [*s*, *t*]. By assumption and Minskowski inequality we have

$$\begin{aligned} \left( \sum _{i=0}^n|x_{s_{i+1}}-x_{s_i}|^p\right) ^{1/p}\le & {} \left[ \sum _{i=0}^n\left( \sum _{j=1}^k C_j \overline{\omega }^j(s_i,s_{i+1})^{1/p}\right) ^p\right] ^{1/p}\\\le & {} \sum _{j=1}^k\left( \sum _{i=0}^n C_j^p\overline{\omega }^j(s_i,s_{i+1})\right) ^{1/p} \le \sum _{j=1}^k C_j \overline{\omega }^j(s,t)^{1/p}. \end{aligned}$$

This implies the conclusion of the lemma. $\square$

Now, consider $x\in \widehat{C}^{q}([a,b],\mathbb {R}^{d\times m})$ and $\omega \in \widehat{C}^p([a,b],\mathbb {R}^m)$, $p,q \ge 1$, if Riemann-Stieltjes sums for finite partition $\Pi =\{ a=t_0<t_1<\cdots < t_n=b \}$ of [*a*, *b*] and any $\xi _i \in [t_i,t_{i+1}]$

2.5 $$\begin{aligned} S_\Pi := \sum _{i=0}^n x_{\xi _i}(\omega _{t_{i+1}}-\omega _{t_i}), \end{aligned}$$

converges as the mesh $|\Pi | := \displaystyle \max \nolimits _{0\le i \le n-1} |t_{i+1}-t_i|$ tends to zero, we call the limit is the *Young integral* of *x* w.r.t $\omega$ on [*a*, *b*] denoted by $\int _a^b x_td\omega _t$. It is well known that if $p,q\ge 1$ and $\frac{1}{p}+\frac{1}{q} > 1$, the Young integral $\int _a^bx_td\omega _t$ exists (see [23, pp. 264–265]). Moreover, if $x^n$ and $\omega ^n$ are of bounded variation, uniformly bounded in $\widehat{C}^q([a,b],\mathbb {R}^{d\times m})$, $\widehat{C}^p([a,b],\mathbb {R}^{m})$ and converges uniformly to *x*, $\omega$ respectively, then the sequence of the Riemann-Stieljes integral $\int _a^b x^n_td\omega ^n_t$ approach $\int _a^bx_td\omega _t$ as $n\rightarrow \infty$ (see [8]). This integral satisfies additive property by the construction, and the so-called Young-Loeve estimate [8, Theorem 6.8, p. 116]

2.6 $$\begin{aligned} \Big |\int _s^t x_ud\omega _u-x_s[\omega _t-\omega _s]\Big | \le K \left| \left| \left| x\right| \right| \right| _{q\text {-var},[s,t]} \left| \left| \left| \omega \right| \right| \right| _{p\text {-var},[s,t]}, \end{aligned}$$

where

2.7 $$\begin{aligned} K:=\left( 1-2^{1-\theta }\right) ^{-1},\qquad \theta := \frac{1}{p} + \frac{1}{q} >1. \end{aligned}$$

#### Lemma 2.4

For $1\le p, 1\le q$ such that $\theta = \frac{1}{p}+\frac{1}{q}>1$ and $x\in \widehat{C}^{q}([a,b],\mathbb {R}^{d\times m})$, $\omega \in \widehat{C}^p([a,b],\mathbb {R}^m)$, the following estimate holds

2.8 $$\begin{aligned} \left| \left| \left| \int _a^.x_ud\omega _u\right| \right| \right| _{p\text {-}\mathrm{{var}},[a,b]}\le & {} \left| \left| \left| \omega \right| \right| \right| _{p\text {-}\mathrm{{var}},[a,b]}\left( |x_a|+(K+1)\left| \left| \left| x\right| \right| \right| _{q\text {-var},[a,b]}\right) , \end{aligned}$$

where *K* is determined by (2.7).

#### Proof

The conclusion is a direct sequence of (2.6) and [8, Proposition 5.10(i), p. 83]. $\square$

Due to Lemma 2.4, the integral $t\mapsto \int _a^tx_sd\omega _s$ is a continuous bounded *p*-variation path. Note that the definition of Young integral does depend on the direction of integration in a simple way like the Riemann-Stieltjes integral. Namely, it is easy to see that

2.9 $$\begin{aligned} \int _b^ax_ud\omega _u= & {} \displaystyle \lim _{\Pi (a,b), |\Pi |\rightarrow 0} \sum _{i=1}^n x_{\xi _i}(\omega _{t_{i}}-\omega _{t_{i+1}}) \nonumber \\= & {} - \displaystyle \lim _{\Pi (a,b), |\Pi |\rightarrow 0} \sum _{i=1}^n x_{\xi _i}(\omega _{t_{i+1}}-\omega _{t_i}) = -\int _a^bx_ud\omega _u. \end{aligned}$$

### The Greedy Sequence of Times

The original idea of a greedy sequence was introduced in [4, Definition 4.7]. Given $\alpha >0$, a compact interval $I \in \mathbb {R}$ and a control $\overline{\omega }: \Delta (I) \rightarrow \mathbb {R}^+$, the construction of such a sequence aims to have a “greedy” approximation to the supremum in the definition of the so-called *accummulated* $\alpha$ *-local* $\overline{\omega }$ *-variation* (see [4, Definition 4.1])

$$\begin{aligned} M_{\alpha ,I}(\overline{\omega }) = \sup _{\Pi (I), \overline{\omega }_{t_i,t_{i+1}} \le \alpha } \sum _{t_i \in \Pi (I)} \overline{\omega }_{t_i,t_{i+1}}. \end{aligned}$$

In particular, $\overline{\omega }_{s,t}$ is chosen to be $\left| \left| \left| \cdot \right| \right| \right| ^p_{p\text {-var},[s,t]}$ in [4].

A similar version for stopping times was developed before in [9] and then has been studied further recently by [7] for stability of the system. Here we propose another version of greedy sequence of times which matches with the nonautonomous setting.

Denote by $\widetilde{C}^{p}(\mathbb {R},\mathbb {R}^m)$ the space of all continuous functions $\omega : \mathbb {R}\rightarrow \mathbb {R}^m$ such that for any $T>0$ the restrictions of $\omega$ to $[-T,T]$ is of $\widehat{C}^{p}([-T,T],\mathbb {R}^m)$. Equip $\widetilde{C}^{p}(\mathbb {R},\mathbb {R}^m)$ with the metric

$$\begin{aligned} d(\omega ^1,\omega ^2) := \sum _{n=1}^\infty 2^{-n} \frac{\Vert \omega ^1 - \omega ^2\Vert _{p\text {-var},[-n,n]}}{1+\Vert \omega ^1 - \omega ^2\Vert _{p\text {-var},[-n,n]}}. \end{aligned}$$

Let $n\in \mathbb {N}$, observe that metric *d* satisfies

2.10 $$\begin{aligned} \begin{array}{rcl} d(\omega ^1,\omega ^2) &{}\le &{} \Vert \omega _1 - \omega ^2\Vert _{p\text {-var},[-n,n]} + 2^{-n},\\ \Vert \omega ^1 -\omega ^2\Vert _{p\text {-var},[-n,n]} &{}\le &{} \frac{2^n d(\omega ^1,\omega ^2)}{1- 2^n d(\omega ^1,\omega ^2)}, \end{array} \end{aligned}$$

where the second inequality holds for any fixed *n* and $\omega ^1, \omega ^2$ close enough such that $2^n d(\omega ^1,\omega ^2) <1$. Hence every Cauchy sequence $(\omega ^k)_k$ w.r.t. metric *d* is also a Cauchy sequence when restricted to $\widehat{C}^p([-n,n],\mathbb {R}^m)$, thus converges to a limit $\omega ^*\in \widehat{C}^p([-n,n],\mathbb {R}^m)$ which is uniquely defined pointwise, so $\omega ^* \in \widetilde{C}^{p}(\mathbb {R},\mathbb {R}^m)$. Therefore, $(\widetilde{C}^{p}(\mathbb {R},\mathbb {R}^m),d)$ is a complete metric space.

#### Remark 2.5

(i) *Truncation:* Another consequence of (2.10) is that the truncated version of $\omega \in \widetilde{C}^{p}(\mathbb {R},\mathbb {R}^m)$ in any $\widehat{C}^p([-n,n],\mathbb {R}^m)$ differs very little w.r.t. metric *d* from the original $\omega$ if we choose *n* large enough. Moreover, if a function is continuous w.r.t. $\omega$ on any restriction in $\widehat{C}^p([-n,n],\mathbb {R}^m)$ for any $n >0$ then it is also continuous w.r.t. $\omega$ in $\widetilde{C}^{p}(\mathbb {R},\mathbb {R}^m)$ with respect to metric *d*.
(ii) *Concatenation:* Let $a<b<c$. Suppose that $\omega ^1 \in \widehat{C}^{p}([a,b],\mathbb {R}^m)$, $\omega ^2 \in \widehat{C}^{p}([b,c],\mathbb {R}^m)$ and $\omega ^1_b=\omega ^2_b$. Then $\omega ^1. 1_{[a,b]} +\omega ^2.1_{[b,c]}$ belongs to $\widehat{C}^{p}([a,c],\mathbb {R}^m)$.

For any given $\lambda ,\mu >0$ we construct a strict increasing sequence of times $\{\tau _n\}$,

$$\begin{aligned} \tau _n:\widetilde{C}^{p}(\mathbb {R},\mathbb {R}^m)\longrightarrow \mathbb {R}^+, \end{aligned}$$

such that $\tau _0\equiv 0$ and

2.11 $$\begin{aligned} |\tau _{i+1}(\omega )-\tau _i(\omega )|^\lambda +\left| \left| \left| \omega \right| \right| \right| _{p\text {-var},[\tau _i(\omega ),\tau _{i+1}(\omega )]} = \mu . \end{aligned}$$

To do so, first define $\tau : \widetilde{C}^{p}(\mathbb {R},\mathbb {R}^m)\longrightarrow \mathbb {R}^+$ such that

$$\begin{aligned} \tau (\omega ) := \sup \{t\ge 0: t^\lambda + \left| \left| \left| \omega \right| \right| \right| _{p\text {-var},[0,t]} \le \mu \}. \end{aligned}$$

Observe that the function $\kappa (t):= t^\lambda + \left| \left| \left| \omega \right| \right| \right| _{p\text {-var},[0,t]}$ is continuous and stricly increasing w.r.t. *t* with $\kappa (0) =0$ and $\kappa (\infty ) = \infty$, therefore due to the continuity there exists a unique $\tau = \tau (\omega )>0$ such that

2.12 $$\begin{aligned} \tau ^\lambda + \left| \left| \left| \omega \right| \right| \right| _{p\text {-var},[0,\tau ]} = \mu . \end{aligned}$$

Thus $\tau$ is well defined. Next, we construct the time sequence inductively as follows. Set $\tau _0 := 0$, $\tau _1 (\omega ) := \tau (\omega )$. Suppose that we have defined $\tau _n (\omega )$ for $n\ge 1$, looking at the following equality as an equation of $\delta _n(\omega )\in \mathbb {R}^+$, like above we find an unique $\delta _n(\omega )$ such that

$$\begin{aligned} \mu = \delta _n^\lambda (\omega ) + \left| \left| \left| \omega (\cdot + \tau _n(\omega ))\right| \right| \right| _{p-var,[0,\delta _n(\omega )]}, \end{aligned}$$

hence we can set

2.13 $$\begin{aligned} \tau _{n+1}(\omega ) := \tau _{n-1}(\omega ) + \delta _n(\omega ), \end{aligned}$$

where $\delta _n(\omega )$ is determined above. Thus we have defined a time sequence $\{\tau _n\}$ for all $n=0,1,2,\ldots$. Such a sequence then satisfies (2.11).

Now, we fix $\omega \in \widetilde{C}^{p}(\mathbb {R},\mathbb {R}^m)$ and consider the number of times of the greedy sequence inside an arbitrary finite interval of $\mathbb {R}^+$. We write $\tau _n$ for $\tau _n(\omega )$ to simplify the notation. For given $T>0$, we introduce the notation

2.14 $$\begin{aligned} N(T,\omega ):= \sup \{n: \tau _n\le T\}<\infty . \end{aligned}$$

or more generally, for any $0\le a< b <\infty$,

2.15 $$\begin{aligned} N(a,b,\omega ):= & {} \sup \{n: \tau _n\le b\}- \inf \{n: \tau _n\ge a\}. \end{aligned}$$

#### Lemma 2.6

Let $p'\ge \max \{p,\frac{1}{\lambda }\}$ be arbitrary, the following estimate holds

2.16 $$\begin{aligned} N(T,\omega )\le \frac{2^{p'-1}}{\mu ^{p'}} \Big ( T^{p'\lambda }+\left| \left| \left| \omega \right| \right| \right| ^{p'}_{p\text {-var},[0,T]}\Big ). \end{aligned}$$

More generally,

2.17 $$\begin{aligned} N(a,b,\omega )\le \frac{2^{p'-1}}{\mu ^{p'} }\Big [ (b-a)^{p'\lambda }+\left| \left| \left| \omega \right| \right| \right| ^{p'}_{p\text {-}\mathrm{var},[a,b]}\Big ]. \end{aligned}$$

#### Proof

We have for all $n\in \mathbb {N}^*$

2.18 $$\begin{aligned} n\mu ^{p'}= & {} \sum _{i=0}^{n-1} \mu ^{p'} = \sum _{i=0}^{n-1}\left[ |\tau _{i+1}-\tau _i|^{\lambda }+\left| \left| \left| \omega \right| \right| \right| _{p\text {-var},[\tau _i,\tau _{i+1}]}\right] ^{p'} \nonumber \\\le & {} 2^{p'-1}\left[ \sum _{i=0}^{n-1} |\tau _{i+1}-\tau _i|^{p'\lambda }+ \left( \sum _{i=0}^{n-1} \left| \left| \left| \omega \right| \right| \right| ^p_{p\text {-var},[\tau _i,\tau _{i+1}]}\right) ^{p'/p}\right] \nonumber \\\le & {} 2^{p'-1}\left[ (\tau _n-\tau _0)^{p'\lambda }+ \left( \sum _{i=0}^{n-1} \left| \left| \left| \omega \right| \right| \right| ^p_{p\text {-var},[\tau _i,\tau _{i+1}]}\right) ^{p'/p}\right] \nonumber \\\le & {} 2^{p'-1}\left[ \tau _n^{p'\lambda } + \left| \left| \left| \omega \right| \right| \right| ^{p'}_{p\text {-var},[0,\tau _n]}\right] . \end{aligned}$$

Consequently, we obtain

$$\begin{aligned} N(T,\omega )\le \frac{2^{p'-1}}{\mu ^{p'}} \Big [ T^{p'\lambda }+\left| \left| \left| \omega \right| \right| \right| ^{p'}_{p\text {-var},[0,T]}\Big ]. \end{aligned}$$

Similarly, (2.17) holds. $$\square $$

#### Remark 2.7

(i) Since the left-hand side of (2.18) tends to infinity as *n* goes to $\infty$ its right hand side can not be bounded. This implies that $\tau _n\rightarrow \infty$ as $n\rightarrow \infty$.
(ii) We can construct a time sequence starts at $\tau _0=t_0$, an arbitrary point in $\mathbb {R}$, and on $(-\infty , t_0]$ in a similar manner.

## Existence and Uniqueness Theorem

In this section, we are working with the restriction of any trajectory $\omega$ in a given time interval [0, *T*] by considering it as an element in $\widehat{C}^{p}([0,T],\mathbb {R}^m)$, for a certain $p \in (1,2)$ (see Remark 2.5 for the relation between $\omega \in \widetilde{C}^{p}(\mathbb {R},\mathbb {R}^m)$ and its restrictions). Consider the Young differential equation in the integral form as:

3.1 $$\begin{aligned} x_t=x_{0} +\int _0^t f(s,x_s) ds + \int _0^tg(s,x_s)d\omega _s,\;\; t\in [0,T]. \end{aligned}$$

We recall here a result in [21] on existence and uniqueness of solution of (3.1), which was proved using contraction mapping arguments with $\omega$ in a Besov-type space. In this paper we however would like to derive a proof in $\widehat{C}^p$ applying Shauder fixed point theorem and greedy sequence of times tool. First we need to formulate some assumptions on the coefficient functions *f* and *g* of (3.1).

($\mathbf{H }_1$) *g*(*t*, *x*) is differentiable in *x* and there exist some constants $0<\beta ,\delta \le 1$, a control function *h*(*s*, *t*) defined on $\Delta [0,T]$ and for every $N\ge 0$ there exists $M_N>0$ such that the following properties hold:

$$\begin{aligned} (H_g) : {\left\{ \begin{array}{ll} (i)\quad \hbox {Lipschitz continuity}\\ |g(t,x) - g(t,y)| \le L_g |x-y|,\quad \forall x,y \in \mathbb {R}^d, \quad \forall t\in [0,T],\\ (ii) \quad \hbox {Local H}\ddot{\mathrm{o}}\hbox {lder continuity}\\ |\partial _{x_i}g(t,x)- \partial _{y_i}g(t,y)| \le M_N |x-y|^\delta , \\ \quad \forall x,y\in \mathbb {R}^d,\; |x|,|y|\le N,\quad \forall t\in [0,T],\\ (iii) \quad \hbox {Generalized H}\ddot{\mathrm{o}}\hbox {lder continuity in time} \\ |g(t,x)-g(s,x)| + |\partial _{x_i}g(t,x) - \partial _{x_i}g(s,x)| \le h(s,t)^\beta \\ \quad \forall x\in \mathbb {R}^d, \quad \forall s,t\in [0,T], s<t. \end{array}\right. } \end{aligned}$$

($\mathbf{H }_2$) There exists $a>0$ and $b\in L^{\frac{1}{1-\alpha }}([0,T],\mathbb {R}^d)$, where $\frac{1}{2} \le \alpha <1$, and for every $N\ge 0$ there exists $L_N >0$ such that the following properties hold:

$$\begin{aligned} (H_f) : {\left\{ \begin{array}{ll} (i)\quad \hbox {Local Lipschitz continuity}\\ |f(t,x) - f(t,y)| \le L_N |x-y|, \quad \forall x,y\in \mathbb {R}^d,\;|x|,|y|\le N, \quad \forall t\in [0,T],\\ (ii) \quad \hbox {Boundedness}\\ |f(t,x)| \le a |x| + b(t), \quad \forall x\in \mathbb {R}^d,\quad \forall t\in [0,T].\\ \end{array}\right. } \end{aligned}$$

($\mathbf{H }_3$) The parameters in $\mathbf{H }_1$ and $\mathbf{H }_2$ statisfy the inequalities $\delta>p-1,\;\;\beta>1-\dfrac{1}{p},\;\; \delta \alpha > 1-\dfrac{1}{p}$.

We would like to study the existence and uniqueness of the solution of (3.1) under the given conditions that $x\in \widehat{C}^{q}([0,T],\mathbb {R}^d)$ with appropriate constant $q>0$.

By the assumption $p\in (1,2)$ and the condition $\mathbf{H }_3$, $1-\dfrac{1}{p}<\min \left\{ \beta ,\delta \alpha , \dfrac{\delta }{p},\dfrac{1}{2} \right\}$, we can choose consecutively constants $q_0,q$ such that

3.2 $$\begin{aligned} 1-\dfrac{1}{p}< & {} \dfrac{1}{q_0} <\min \left\{ \beta ,\delta \alpha , \dfrac{\delta }{p},\dfrac{1}{2} \right\} , \end{aligned}$$

3.3 $$\begin{aligned} \frac{1}{q_0\delta }\le & {} \dfrac{1}{q} < \min \left\{ \alpha , \dfrac{1}{p}\right\} . \end{aligned}$$

Then, we have

3.4 $$\begin{aligned} \frac{1}{p}+\frac{1}{q_0}> 1,\;\; q_0\beta>1,\;\; q_0\ge q_0\delta \ge q> p,\;\; q\alpha > 1. \end{aligned}$$

We now consider $x\in \widehat{C}^{q}([t_0,t_1],\mathbb {R}^d)$ with some $[t_0,t_1] \subset [0,T]$. Define the mapping given by

3.5 $$\begin{aligned} F(x)_t= & {} x_{t_0} + I(x)_t+J(x)_t \nonumber \\:= & {} x_{t_0} + \int _{t_0}^t f(s,x_s) ds +\int _{t_0}^t g(s,x_s)d\omega _s, \quad \forall t\in [t_0,t_1]. \end{aligned}$$

Note that a fixed point of *F* is a solution of (3.1) on $[t_0,t_1]$ with the boundary condition $x(t_0)=x_{t_0}$ (the initial condition $x_{t_0}$ of (3.1) is then not given).

Introduce the notations

3.6 $$\begin{aligned} M:= & {} \max \left\{ L_g,aT^{1-\alpha },|g(0,0)|+h(0,T)^\beta ,\Vert b\Vert _{L^{\frac{1}{1-\alpha }}}\right\} , \end{aligned}$$

3.7 $$\begin{aligned} M'_N:= & {} \max \{L_N,M_N,M\},\qquad \forall N>0. \end{aligned}$$

It can be seen from the above assumptions that $|g(t,x)|\le |g(t,0)|+ L_g|x|$ and $|g(t,0)|\le |g(0,0)|+ h(0,T)^{\beta }$, hence

3.8 $$\begin{aligned} |g(t,x)|\le |g(0,0)|+ h(0,T)^{\beta }+ L_g|x| \le M(1+|x|). \end{aligned}$$

For the next proposition we need the following auxiliary lemma.

### Lemma 3.1

Assume that $\mathbf{H }_1-\mathbf{H }_3$ are satisfied.

(i) If $x \in \widehat{C}^{q}([t_0,t_1],\mathbb {R}^d)$ then $g(\cdot ,x_.) \in \widehat{C}^{q_0}([t_0,t_1],\mathbb {R}^{d\times m})$ and
  3.9 $$\begin{aligned} \left| \left| \left| g(\cdot ,x_.)\right| \right| \right| _{q_0\text {-}\mathrm{var},[t_0,t_1]}\le M(1+\left| \left| \left| x\right| \right| \right| _{q\text {-}\mathrm{var},[t_0,t_1]}). \end{aligned}$$
(ii) For all $s< t$ and for all $x_i\in \mathbb {R}^d$ such that $|x_i|\le N$, $i=1,2,3,4$, then
  $$\begin{aligned} |g(s,x_1)-g(s,x_3)-g(t,x_2)+g(t,x_4)|\le & {} L_g|x_1-x_2-x_3+x_4|+ |x_2-x_4|h(s,t)^\beta \\&+\,M_N|x_2-x_4|(|x_1-x_2|^{\delta }+|x_3-x_4|^{\delta }). \end{aligned}$$
(iii) For any $x,y \in \widehat{C}^{q}([t_0,t_1],\mathbb {R}^d)$ such that $x_{t_0} = y_{t_0}$ and $\Vert x\Vert _{\infty ,[t_0,t_1]}\le N$, $\Vert y\Vert _{\infty ,[t_0,t_1]}\le N$ we have
  3.10 $$\begin{aligned} \left| \left| \left| g(\cdot ,x_.)-g(\cdot ,y_.)\right| \right| \right| _{q_0\text {-}\mathrm{var},[t_0,t_{1}]}\le & {} M'_N\left| \left| \left| x-y\right| \right| \right| _{q\text {-}\mathrm{var},[t_0,t_{1}]} \nonumber \\&\left( 2+\left| \left| \left| x\right| \right| \right| ^{\delta }_{q\text {-}\mathrm{var},[t_0,t_{1}]}+\left| \left| \left| y\right| \right| \right| ^{\delta }_{q\text {-}\mathrm{var},[t_0,t_{1}]}\right) .\nonumber \\ \end{aligned}$$

### Proof

(i) For $s<t$ in $[t_0,t_1]$, we have

$$\begin{aligned} |g(t,x_t)-g(s,x_s)|\le & {} |g(t,x_t)-g(t,x_s)|+|g(t,x_s)-g(s,x_s)|\\\le & {} L_g|x_t-x_s| + h(s,t)^{\beta }. \end{aligned}$$

Let $\Pi =(s_i)_1^{n+1}$ be an arbitrary finite partition of $[t_0,t_1]$, $s_1=t_0, s_{n+1}=t_1$. Since $q_0\ge q$ and $q_0\beta >1$ we have

$$\begin{aligned} \left( \sum _{i=1}^{n}|g(s_{i+1},x_{s_{i+1}})-g({s_i},x_{s_i})|^{q_0}\right) ^{1/q_0}\le & {} L_g \left( \sum _{i=1}^{n} |x_{s_{i+1}}-x_{s_i}|^{q_0}\right) ^{1/q_0} \\&+ \left( \sum _{i=1}^{n} h(s_{i},s_{i+1})^{q_0\beta } \right) ^{1/q_0}\\\le & {} L_g\left| \left| \left| x\right| \right| \right| _{q_0\text {-var},[t_0,t_1]}+ h(t_0,t_1)^{\beta }\\\le & {} L_g\left| \left| \left| x\right| \right| \right| _{q\text {-var},[t_0,t_1]}+ h(0,T)^{\beta } \\\le & {} M(1+\left| \left| \left| x\right| \right| \right| _{q\text {-var},[t_0,t_1]})<\infty . \end{aligned}$$

Take the superemum over the set of all finite partition $\Pi$ we get $g(\cdot ,x_.) \in \widehat{C}^{q_0}([t_0,t_1],\mathbb {R}^{d\times m})$ and

$$\begin{aligned} \left| \left| \left| g(\cdot ,x_.)\right| \right| \right| _{q_0\text {-var},[t_0,t_1]}\le M(1+\left| \left| \left| x\right| \right| \right| _{q\text {-var},[t_0,t_1]}). \end{aligned}$$

(ii) This part is similar to [21, Lemma 7.1] with our function $h(s,t)^\beta$ playing the role of $|t-s|^\beta$ in [21, Lemma 7.1].

(iii) Note that $q_0\beta >1$ and $q_0\delta \ge q$ hence

$$\begin{aligned}&\left| \left| \left| g(\cdot ,x_.)-g(\cdot ,y_.)\right| \right| \right| _{q_0\text {-var},[t_0,t_{1}]}\\&\quad := \left( \sup _{\Pi ([t_0,t_1])} \sum _{i} | g(s_{i+1},x_{s_{i+1}})- g(s_{i+1},y_{s_{i+1}}) -g(s_i,x_{s_{i}}) +g(s_{i},y_{s_{i}}) |^{q_0}\right) ^{1/q_0}\nonumber \\&\quad \le L_g\sup _{\Pi ([t_0,t_1])}\left( \sum _i |x_{s_{i+1}}-y_{s_{i+1}}-x_{s_i}+y_{s_i} |^{q_0}\right) ^{1/q_0}\\&\qquad +\, \Vert x-y\Vert _{\infty ,[t_0,t_1]}\sup _{\Pi ([t_0,t_1])}\left( \sum _i h(s_{i},s_{i+1})^{q_0\beta }\right) ^{1/q_0}\nonumber \\&\qquad +\, M_N \Vert x-y\Vert _{\infty ,[t_0,t_1]} \sup _{\Pi ([t_0,t_1])} \left[ \left( \sum _i |x_{s_{i+1}}-x_{s_i}|^{q_0\delta }\right) ^{1/q_0}\right. \nonumber \\&\qquad \left. +\left( \sum _i |y_{s_{i+1}}-y_{s_i}|^{q_0\delta }\right) ^{1/q_0}\right] \nonumber \\&\quad \le L_g\left| \left| \left| x-y\right| \right| \right| _{q\text {-var},[t_0,t_1]}+ \Vert x-y\Vert _{\infty ,[t_0,t_1]}\nonumber \\&\qquad \times \left[ h(t_0,t_1)^{\beta } + M_N \left( \left| \left| \left| x\right| \right| \right| ^{\delta }_{q\text {-var},[t_0,t_{1}]}+\left| \left| \left| y\right| \right| \right| ^{\delta }_{q\text {-var},[t_0,t_{1}]}\right) \right] \nonumber \\&\quad \le M'_N\left| \left| \left| x-y\right| \right| \right| _{q\text {-var},[t_0,t_{1}]} \left( 2+\left| \left| \left| x\right| \right| \right| ^{\delta }_{q\text {-var},[t_0,t_{1}]}+\left| \left| \left| y\right| \right| \right| ^{\delta }_{q\text {-var},[t_0,t_{1}]}\right) . \end{aligned}$$

The lemma is proved. $\square$

For a proof of our main theorem on existence and uniqueness of solutions of an Young differential equation, we need the following proposition.

### Proposition 3.2

Assume that $\mathbf{H }_1-\mathbf{H }_3$ are satisfied. Let $0\le t_0<t_1\le T$ be arbitrary, *q* be chosen as above satisfying (3.3) and *F* be defined by (3.5). Then for any $x\in \widehat{C}^{q}([t_0,t_1],\mathbb {R}^d)$ we have $F(x)\in \widehat{C}^{q}([t_0,t_1],\mathbb {R}^d)$, thus

$$\begin{aligned} F: \widehat{C}^{q}([t_0,t_1],\mathbb {R}^d)\longrightarrow \widehat{C}^{q}([t_0,t_1],\mathbb {R}^d). \end{aligned}$$

Moreover, the following statements hold

(i) The *q*-variation of *F*(*x*) satisfies
  3.11 $$\begin{aligned} \left| \left| \left| F(x)\right| \right| \right| _{q\text {-}\mathrm{var},[t_0,t_1]}\le & {} M(K+2)\left( 1+\Vert x\Vert _{q\text {-}\mathrm{var},[t_0,t_1]}\right) \nonumber \\&\times \left( (t_1-t_0)^{\alpha }+\left| \left| \left| \omega \right| \right| \right| _{p\text {-}\mathrm{var},[t_0,t_1]}\right) . \end{aligned}$$
(ii) Let $N\ge 0$ be arbitrary but fixed. Suppose that $x,y\in \widehat{C}^{q}([t_{0},t_{1}],\mathbb {R}^d)$ be such that $\Vert x\Vert _{\infty ,[t_0,t_1]}\le N$, $\Vert y\Vert _{\infty ,[t_0,t_1]}\le N$ and $x_{t_0} = y_{t_0}$, then we have
  3.12 $$\begin{aligned}&\Vert F(x)-F(y)\Vert _{q\text {-}\mathrm{var},[t_0,t_{1}]}\nonumber \\&\quad \le \Vert x-y\Vert _{q\text {-}\mathrm{var},[t_0,t_{1}]}\left( (t_1-t_0)+\left| \left| \left| \omega \right| \right| \right| _{p\text {-}\mathrm{var},[t_0,t_1]}\right) \nonumber \\&\qquad \times M'_N (K+1) \left( 2+\left| \left| \left| x\right| \right| \right| ^{\delta }_{q\text {-}\mathrm{var},[t_0,t_{1}]}+\left| \left| \left| y\right| \right| \right| ^{\delta }_{q\text {-}\mathrm{var},[t_0,t_{1}]}\right) . \end{aligned}$$

### Proof

(i) Since $\frac{1}{p}+\frac{1}{q_0} > 1$, by virtue of (3.9), the Young integral $\int _0^tg(s,x_s)d\omega _s$ exists for all $t\in [t_0,t_1]$. Using (2.8), (3.5) and (3.8) we get

$$\begin{aligned} \left| \left| \left| J(x)\right| \right| \right| _{q\text {-var},[t_0,t_1]}\le & {} \left| \left| \left| \omega \right| \right| \right| _{p\text {-var},[t_0,t_1]}\left[ |g(t_0,x_{t_0})|+ (K+1) \left| \left| \left| g(.,x_.)\right| \right| \right| _{q_0\text {-var},[t_0,t_1]}\right] \nonumber \\\le & {} \left| \left| \left| \omega \right| \right| \right| _{p\text {-var},[t_0,t_1]}\left[ M(1+|x_{t_0}|)+M(K+1)(1+\left| \left| \left| x\right| \right| \right| _{q\text {-var},[t_0,t_1]})\right] \\\le & {} \left| \left| \left| \omega \right| \right| \right| _{p\text {-var},[t_0,t_1]}M\left[ (K+2) + |x_{t_0}| +(K+1)\left| \left| \left| x\right| \right| \right| _{q\text {-var},[t_0,t_1]} \right] . \end{aligned}$$

Now, by Hölder inequality and the assumption $\mathbf{H }_2$ we have

$$\begin{aligned} \int _s^t |b(u)|du\le \left( \int _s^t|b(u)|^\frac{1}{1-\alpha } du \right) ^{1-\alpha } \left( \int _s^t 1 du \right) ^\alpha \le \Vert b\Vert _{L^{\frac{1}{1-\alpha }}} (t-s)^{\alpha }\le M(t-s)^{\alpha }. \end{aligned}$$

Therefore, for $s<t$ in $[t_0,t_1]$ using the assumption $\mathbf{H }_2$ we have

$$\begin{aligned} \left| \int _s^tf(u,x_u)du\right|\le & {} a\Vert x\Vert _{\infty ,[s,t]}(t-s) + \Vert b\Vert _{L^{\frac{1}{1-\alpha }}} (t-s)^{\alpha }\\\le & {} (t-s)^{\alpha } \left( aT^{1-\alpha }\Vert x\Vert _{\infty ,[t_0,t_1]} + \Vert b\Vert _{L^{\frac{1}{1-\alpha }}}\right) \\\le & {} (t-s)^{\alpha }M \left( 1+|x_{t_0}|+\left| \left| \left| x\right| \right| \right| _{q\text {-var},[t_0,t_1]}\right) . \end{aligned}$$

This implies

$$\begin{aligned} \left| \left| \left| I(x)\right| \right| \right| _{q\text {-var},[t_0,t_1]}=\left| \left| \left| \int _{t_0}^.f(u,x_u)du\right| \right| \right| _{q\text {-var},[t_0,t_1]}\le M(t_1-t_0)^{\alpha } \left( 1+|x_{t_0}|+\left| \left| \left| x\right| \right| \right| _{q\text {-var},[t_0,t_1]}\right) \end{aligned}$$

by [8, Proposition 5.10(i), p. 83] and the fact that the function $(s,t)\rightarrow (t-s)^{q\alpha }$ defined on $\Delta [t_0,t_1]$ is a control function for $q\alpha > 1$. Since

$$\begin{aligned} \left| \left| \left| F(x)\right| \right| \right| _{q\text {-var},[t_0,t_1]}\le \left| \left| \left| I(x)\right| \right| \right| _{q\text {-var},[t_0,t_1]}+\left| \left| \left| J(x)\right| \right| \right| _{q\text {-var},[t_0,t_1]} \end{aligned}$$

(3.11) holds.

(ii) By virtue of (2.8), (3.10) and the condition $x_{t_0} =y_{t_0}$, we have

$$\begin{aligned} \left| \left| \left| J(x)-J(y)\right| \right| \right| _{p\text {-var},[t_0,t_{1}]}\le & {} \left| \left| \left| \omega \right| \right| \right| _{p\text {-var},[t_0,t_1]}\left[ |g(t_0,x_{t_0}) - g(t_0,y_{t_0})|\right. \\&\left. +\, (K+1)\left| \left| \left| g(.,x_.) -g(.,y_.)\right| \right| \right| _{q_0\text {-var},[t_0,t_1]}\right] \\\le & {} \left| \left| \left| \omega \right| \right| \right| _{p\text {-var},[s,t]}(K+1)\left| \left| \left| g(.,x_.) - g(.,y_.)\right| \right| \right| _{q_0\text {-var},[t_0,t_1]}\\\le & {} (K+1)M'_N\left| \left| \left| \omega \right| \right| \right| _{p\text {-var},[t_0,t_1]}\Vert x-y\Vert _{q\text {-var},[t_0,t_{1}]}\\&\times \left( 2+\left| \left| \left| x\right| \right| \right| ^{\delta }_{q\text {-var},[t_0,t_{1}]}+\left| \left| \left| y\right| \right| \right| ^{\delta }_{q\text {-var},[t_0,t_{1}]}\right) . \end{aligned}$$

Similarly,

$$\begin{aligned} \left| [I(x)_t-I(y)_t]- [I(x)_s-I(y)_s]\right|\le & {} \int _s^t |f(u,x_u)-f(u,y_u)|du \\\le & {} L_N \Vert x-y\Vert _{q\text {-var},[t_0,t_1]} (t-s), \end{aligned}$$

hence

$$\begin{aligned} \left| \left| \left| I(x)-I(y)\right| \right| \right| _{q\text {-var},[t_0,t_1]} \le M'_N \Vert x-y\Vert _{q\text {-var},[t_0,t_1]} (t_1-t_0). \end{aligned}$$

Inequality (3.12) is a direct consequence of these estimates for *I*(*x*) and *J*(*x*). $\square$

Before proving the existence and uniqueness theorem, we need the following lemma of Gronwall type.

### Lemma 3.3

*(Gronwall-type Lemma)* Let $1\le p\le q$ be arbitrary and satisfy $\frac{1}{p}+\frac{1}{q}>1$. Assume that $\omega \in \widehat{C}^p([0,T],\mathbb {R})$ and $y\in \widehat{C}^{q}([0,T],\mathbb {R}^d)$ satisfy

3.13 $$\begin{aligned} |y_t-y_s|\le A_{s,t}^{1/q} + a_1\left| \int _s^t y_udu \right| + a_2\left| \int _s^t y_ud\omega _u\right| ,\quad \forall s,t\in [0,T],\quad s<t, \end{aligned}$$

for some fixed control function *A* on $\Delta [0,T]$ and some constants $a_1, a_2 \ge 0$. Then there exists a constant *C* independent of *T* such that for every $s,t \in [0,T]$, $s<t$,

3.14 $$\begin{aligned} \left| \left| \left| y\right| \right| \right| _{q\text {-}\mathrm{var},[s,t]} \le (|y_s| + A_0) e^{C(|t-s|^p+ \left| \left| \left| \omega \right| \right| \right| _{p\text {-}\mathrm{var},[s,t]}^{p})}, \end{aligned}$$

where $$A_0 =A_{0,T}^{1/q}$$.

### Proof

Put

$$\begin{aligned} c:=\max \{a_1, a_2(K+1)\}, \end{aligned}$$

in which *K* is defined in (2.7). We have

$$\begin{aligned} |y_t-y_s|\le & {} A_{s,t}^{1/q}+a_1\Vert y\Vert _{\infty ,[s,t]}(t-s) + a_2\left| \left| \left| \omega \right| \right| \right| _{p\text {-var},[s,t]}(\Vert y\Vert _{\infty ,[s,t]} + K\left| \left| \left| y\right| \right| \right| _{q\text {-var},[s,t]})\\\le & {} A_{s,t}^{1/q}+\max \left\{ a_1\Vert y\Vert _{\infty ,[s,t]}, a_2(\Vert y\Vert _{\infty ,[s,t]}\right. \\&\left. +\,K\left| \left| \left| y\right| \right| \right| _{q\text {-var},[s,t]})\right\} (t-s+\left| \left| \left| \omega \right| \right| \right| _{p\text {-var},[s,t]}). \end{aligned}$$

Fix the interval $[s,t]\subset [0,T]$ and apply the above inequality for arbitrary subinterval $[u,v]\subset [s,t]$ we obtain

$$\begin{aligned} |y_v-y_u|\le & {} A_{u,v}^{1/q}+\max \{a_1\Vert y\Vert _{\infty ,[s,t]}, a_2(\Vert y\Vert _{\infty ,[s,t]}\\&+\,K\left| \left| \left| y\right| \right| \right| _{q\text {-var},[s,t]})\} (v-u+\left| \left| \left| \omega \right| \right| \right| _{p\text {-var},[u,v]})\\\le & {} A_{u,v}^{1/q}+\max \{a_1, a_2(K+1)\}(|y_s| +\left| \left| \left| y\right| \right| \right| _{q\text {-var},[s,t]}) (v-u+\left| \left| \left| \omega \right| \right| \right| _{p\text {-var},[u,v]})\\\le & {} A_{u,v}^{1/q}+c(|y_s| +\left| \left| \left| y\right| \right| \right| _{q\text {-var},[s,t]}) (v-u+\left| \left| \left| \omega \right| \right| \right| _{p\text {-var},[u,v]}). \end{aligned}$$

Therefore, by virtue of Lemma 2.3, we get

3.15 $$\begin{aligned} \left| \left| \left| y\right| \right| \right| _{q\text {-var},[s,t]}\le & {} A_{s,t}^{1/q}+ c(|y_s| +\left| \left| \left| y\right| \right| \right| _{q\text {-var},[s,t]}) (t-s+\left| \left| \left| \omega \right| \right| \right| _{p\text {-var},[s,t]})\nonumber \\\le & {} A_0+ c(|y_s| +\left| \left| \left| y\right| \right| \right| _{q\text {-var},[s,t]}) (t-s+\left| \left| \left| \omega \right| \right| \right| _{p\text {-var},[s,t]}). \end{aligned}$$

Now we construct the time sequence $t_i$ with parameter $\{1, \frac{1}{2c}\}$ according to Sect. 2.2, that is

$$\begin{aligned} (t_{i+1}-t_i+\left| \left| \left| \omega \right| \right| \right| _{p\text {-var},[t_i,t_{i+1}]})=\frac{1}{2c}. \end{aligned}$$

Then, by (3.15) for all $s,t \in [t_i,t_{i+1}]$, $s<t$, we have $\left| \left| \left| y\right| \right| \right| _{q\text {-var},[s,t]} \le A_0 + \frac{1}{2}(|y_s| + \left| \left| \left| y\right| \right| \right| _{q\text {-var},[s,t]}),$ which implies $\left| \left| \left| y\right| \right| \right| _{q\text {-var},[u,v]}\le 2A_0+ |y_{u}|$ for all $u,v \in [t_i,t_{i+1}],\; u<v$. Therefore, $|y_{t_{i+1}}|\le \Vert y\Vert _{\infty ,[s,t_{i+1}]}\le 2(A_0+|y_{s}|)$ for all $s\in [t_i,t_{i+1}]$. By induction we obtain for any $s\in [t_k,t_{k+1}]$, $0\le k\le i$, $i\in \{0,\dots , N(T,\omega )\}$, where $N(T,\omega )$ is defined by (2.14), the sequence of inequalities

$$\begin{aligned} 2A_0+ | y_{t_{i+1}}|\le 2(2A_0+ |y_{t_i}|)\le \dots \le 2^{i-k}(2A_0+|y_{t_{k+1}}|)\le 2^{i-k+1}(2A_0+|y_{s}|). \end{aligned}$$

Hence,

3.16 $$\begin{aligned} \left| \left| \left| y\right| \right| \right| _{q\text {-var},[t_i,t_{i+1}]} \le 2A_0+ | y_{t_{i}}|\le 2^{i-k}(2A_0+|y_{s}|),\quad \forall s\in [t_k,t_{k+1}],\;0\le k\le i.\nonumber \\ \end{aligned}$$

Now, we estimate the *q*-var norm of *y* in an arbitrary but fixed interval $[s,t]\subset [0,T]$. Recall the time sequence defined in (2.11). If there exists *i* such that $s<t_i<t$, put

$$\begin{aligned} \overline{N}:= \sup \{n: t_n\le t\},\;\; \underline{N}:= \inf \{n: t_n\ge s\}, \;\; N:= \overline{N}- \underline{N}=N(s,t,\omega ), \end{aligned}$$

where $N(s,t,\omega )$ is defined in (2.15). We have $s\le t_{\underline{N}}<t_{\underline{N}+1}<\cdots < t_{\overline{N}}\le t$ and

$$\begin{aligned} \left| \left| \left| y\right| \right| \right| _{q\text {-var},[s,t_{\underline{N}}]}\le & {} 2A_0 + |y_s|,\\ \left| \left| \left| y\right| \right| \right| _{q\text {-var},[t_{\underline{N}+i},t_{\underline{N}+i+1}]}\le & {} 2^{i+1}( 2A_0 + |y_{s}|), i=0,\dots , N-1,\\ \left| \left| \left| y\right| \right| \right| _{q\text {-var},[t_{\overline{N}},t]}\le & {} 2A_0+|y_{t_{\overline{N}}}| \le 2^{N}( 2A_0 + |y_{s}|). \end{aligned}$$

By Lemma 2.1 we have

$$\begin{aligned} \left| \left| \left| y\right| \right| \right| _{q\text {-var},[s,t]}\le & {} (N+1)^{\frac{q-1}{q}} \left( \left| \left| \left| y\right| \right| \right| ^q_{q\text {-var},[s,t_{\underline{N}}]}+ \sum _{i=1}^{N-1}\left| \left| \left| y\right| \right| \right| ^q_{q\text {-var},[t_i,t_{i+1}]} + \left| \left| \left| y\right| \right| \right| ^q_{q\text {-var},[t_{\overline{N}},t]} \right) ^{1/q}\nonumber \\\le & {} (N+1)^{\frac{q-1}{q}}(2A_0+|y_s|)\left( \sum _{j=0}^{N} 2^{jq}\right) ^{1/q} \nonumber \\\le & {} (N+1)(2A_0+|y_s|)2^{N}. \end{aligned}$$

In the case $[s,t]\subset [t_i,t_{i+1}]$ with some $i\in \{0, 1,\dots , N(T,\omega )\}$, we already have

$$\begin{aligned} \left| \left| \left| y\right| \right| \right| _{q\text {-var},[s,t]}\le 2A_0 + |y_s|. \end{aligned}$$

To sum up, for any $[s,t]\subset [0,T]$ we have the estimate

$$\begin{aligned} \left| \left| \left| y\right| \right| \right| _{q\text {-var},[s,t]}\le (2A_0+|y_s|)2^{2N}. \end{aligned}$$

Combining with (2.17), we conclude that

$$\begin{aligned} \left| \left| \left| y\right| \right| \right| _{q\text {-var},[s,t]}\le & {} (2A_0+|y_s|)2^{4^pc^p(|t-s|^p+\left| \left| \left| \omega \right| \right| \right| ^p_{p\text {-var},[s,t]})}\\\le & {} (2A_0+|y_s|) e^{C(|t-s|^p+ \left| \left| \left| \omega \right| \right| \right| _{p\text {-}\mathrm{var},[s,t]}^{p})}, \end{aligned}$$

where $C= 4^pc^p\ln 2$. The proof is complete. $\square$

### Remark 3.4

(i) Gronwall Lemma is an important tool in the theory of ordinary differential equations, and the theory of Young differential equations as well. Some versions of Gronwall-type lemma can be seen in [21] and [6].
(ii) The conclusion of Lemma 3.3 is still true if one replaces $A_0$ by $A^{1/q}_{s,t}$.
(iii) It can be seen from the proof that in the conditions of Lemma 3.3 we have
  $$\begin{aligned} \Vert y\Vert _{\infty ,[0,T]}\le (2A_0+|y_0|)2^{N(T,\omega )+1}\le (2A_0+|y_0|)2^{(4cT)^p+1+ (4c\left| \left| \left| \omega \right| \right| \right| _{p\text {-var},[s,t]})^p}. \end{aligned}$$

### Corollary 3.5

If in Lemma 3.3 we replace the condition (3.13) by the condition

3.17 $$\begin{aligned} \left| \left| \left| y\right| \right| \right| _{q\text {-}\mathrm{var},[s,t]}\le A^{1/q}_{s,t}+ a_1(|y_s| +\left| \left| \left| y\right| \right| \right| _{q\text {-}\mathrm{var},[s,t]}) (t-s+\left| \left| \left| \omega \right| \right| \right| _{p\text {-}\mathrm{var},[s,t]}) \end{aligned}$$

for all $s<t$ in [0, *T*], a positive constant $a_1>0$ and $\omega \in \widehat{C}^p([0,T],\mathbb {R}^m)$. Then there exists a constant *C* independent of *T* such that for every $s<t$ in [0, *T*]

3.18 $$\begin{aligned} \left| \left| \left| y\right| \right| \right| _{q\text {-}\mathrm{var},[s,t]} \le (|y_s| + A^{1/q}_{s,t})e^{C(|t-s|^p+\left| \left| \left| \omega \right| \right| \right| _{p\text {-}\mathrm{var},[s,t]}^{p})}. \end{aligned}$$

We are now at the position to state and prove the main theorem of this section.

### Theorem 3.6

*(Existence and uniqueness of global solution)* Consider the Young differential equation (3.1), starting from an arbitrary initial time $t_0\in [0,T)$,

$$\begin{aligned} x_t=x_{t_0} +\int _{t_0}^t f(s,x_s) ds + \int _{t_0}^tg(s,x_s)d\omega _s,\quad t\in [t_0,T],\quad x_{t_0}\in \mathbb {R}^d. \end{aligned}$$

with *T* being an arbitrary fixed positive number and $x_0\in \mathbb {R}^d$ being an arbitrary initial condition. Assume that the conditions $\mathbf{H }_1-\mathbf{H }_3$ hold. Then, this equation has a unique solution *x* in the space $\widehat{C}^{q}([t_0,T],\mathbb {R}^d)$, where *q* is chosen as above satisfying (3.3). Moreover, the solution is in $\widehat{C}^{p'}([t_0,T],\mathbb {R}^d)$, where $p'=\max \{p,\frac{1}{\alpha }\}$.

### Proof

The proof proceeds in several steps.

**Step 1**: In this step we will show the *local existence and uniqueness* of solution. Set

3.19 $$\begin{aligned} \mu := \dfrac{1}{2M(K+2)}, \end{aligned}$$

where *M* is defined in (3.6) and *K* is defined in (2.7). Let $s_0\in [t_0,T)$ be arbitrary but fixed. We recall here the time sequence $\tau _n$ with the parameters $\alpha ,\mu$, i.e

$$\begin{aligned} \tau _0 = 0,\;\;|\tau _{i+1}-\tau _i|^\alpha +\left| \left| \left| \omega \right| \right| \right| _{p\text {-var},[\tau _i,\tau _{i+1}]} = \mu . \end{aligned}$$

Put $r_0= \min \{n: \tau _n> s_0\}$ and define $s_1 = \min \{\tau _{r_0}, T\}$. Then,

3.20 $$\begin{aligned} |s_{1}-s_0|^{\alpha }+\left| \left| \left| \omega \right| \right| \right| _{p\text {-var},[s_0,s_{1}]} \le \mu . \end{aligned}$$

We will show that the Eq. (3.1) restricted to $[s_0,s_1]$ has a unique solution.

**Existence of local solutions**.

Recall the mapping *F* defined by the formula (3.5) with $t_0, t_1$ replaced by $s_0,s_1$, respectively. By Proposition 3.2 and (3.19)–(3.20), for $s_0, s_1$ determined above we have $F: \widehat{C}^{q}([s_0,s_1],\mathbb {R}^d)\longrightarrow \widehat{C}^{q}([s_0,s_1],\mathbb {R}^d)$ and

$$\begin{aligned} \Vert F(x)\Vert _{q\text {-var},[s_0,s_1]}= |F(x)_{s_0}|+\left| \left| \left| F(x)\right| \right| \right| _{q\text {-var},[s_0,s_1]} \le |x_{s_0}|+\frac{1}{2}\left( 1+\Vert x\Vert _{q\text {-var},[s_0,s_1]}\right) . \end{aligned}$$

We show furthermore that if $x\in \widehat{C}^{q}([s_0,s_1],\mathbb {R}^d)$ then $F(x)\in C^{(q-\epsilon )\text {-var}}([s_0,s_1],\mathbb {R}^d)$ with small enough $\epsilon$. Indeed, since $q>p$, $q\alpha >1$, we can choose $\epsilon > 0$ such that $q-\epsilon \ge p$ and $(q-\epsilon )\alpha \ge 1$. For all $s<t$ in $[s_0,s_1]$, using (3.11) we have

$$\begin{aligned} |F(x)_t-F(x)_s|\le & {} \left| \left| \left| F(x)\right| \right| \right| _{q\text {-var},[s,t]}\\\le & {} M(K+2)\left( 1+\Vert x\Vert _{q\text {-var},[s_0,s_1]}\right) \left( (t-s)^{\alpha }+\left| \left| \left| \omega \right| \right| \right| _{p\text {-var},[s,t]}\right) \\\le & {} M(K+2)\left( 1+\Vert x\Vert _{q\text {-var},[s_0,s_1]}\right) \\&\times \left[ \left( (t-s)^{(q-\epsilon )\alpha }\right) ^{\frac{1}{q-\epsilon }}+\left( \left| \left| \left| \omega \right| \right| \right| _{p\text {-var},[s,t]}^{(q-\epsilon )}\right) ^{\frac{1}{q-\epsilon }}\right] , \end{aligned}$$

hence

$$\begin{aligned} \left| \left| \left| F(x)\right| \right| \right| _{(q-\epsilon )-\text {var},[s_0,s_1]}\le M(K+2)\left( 1+\Vert x\Vert _{q\text {-var},[s_0,s_1]}\right) \left( (s_1-s_0)^{\alpha }+\left| \left| \left| \omega \right| \right| \right| _{p\text {-var},[s_0,s_1]}\right) \end{aligned}$$

and the assertion follows by an application of Lemma 2.3. Consider the set

$$\begin{aligned} B_1:= \left\{ x\in \widehat{C}^{q}([s_0,s_1],\mathbb {R}^d)|\;x(s_0) = x_{s_0}, \Vert x\Vert _{q\text {-var},[s_0,s_1]}\le 2|x_{s_0}|+1 \right\} . \end{aligned}$$

Taking into account (3.12), $F:B_1 \rightarrow B_1$ is continuous. We show that $B_1$ is a closed convex set in the Banach space $\widehat{C}^{q}([s_0,s_1],\mathbb {R}^d)$, and *F* is a compact operator on $B_1$. Indeed, for the former observation, note that if $z=\lambda x+(1-\lambda )y$ for some $x,y\in B_1, \lambda \in [0,1]$ then

$$\begin{aligned} z_{s_0} = \lambda x_{s_0}+(1-\lambda )y_{s_0}= \lambda x_{s_0}+(1-\lambda )x_{s_0} = x_{s_0} \end{aligned}$$

and

$$\begin{aligned} \Vert z\Vert _{q\text {-var},[s_0,s_1]}= & {} \Vert \lambda x+(1-\lambda )y\Vert _{q\text {-var},[s_0,s_1]}\\\le & {} \lambda \Vert x\Vert _{q\text {-var},[s_0,s_1]}+(1-\lambda )\Vert y\Vert _{q\text {-var},[s_0,s_1]} \le 2|x_{s_0}|+1. \end{aligned}$$

Now, we prove that for any sequence $y^n\in F(B_1)$, there exists an subsequence converges in $p\text {-var}$ norm to an element $y\in B_1$, i.e. $F(B_1)$ is relatively compact in $B_1$. To do that, we will show that $(y^n)$ are equicontinuous, bounded in $(q-\epsilon )\text {-var}$ norm. Namely, take the sequence $y^n=F(x^n)\in F(S)$, $x^n\in B_1$. Then, by virtue of Lemma 2.3 we have

$$\begin{aligned} \sup _n \Vert y^n\Vert _{(q-\epsilon )\text {-var},[s_0,s_1]}\le |x_{s_0}|+ 2M(K+2)(1+|x_{s_0}|)((s_1-s_0)^{\alpha }+\left| \left| \left| \omega \right| \right| \right| _{p\text {-var},[s_0,s_1]}). \end{aligned}$$

It means that $y^n$ are bounded in $C([s_0,s_1],\mathbb {R}^d)$ with sup norm, as well as bounded in $C^{(q-\epsilon )\text {-var}}([s_0,s_1],\mathbb {R}^d)$. Moreover, for all *n*, $s_0\le s\le t\le s_1$,

$$\begin{aligned} |y^n_t-y^n_s|\le 2M(K+2)(1+|x_{s_0}|)\left( (t-s)^{\alpha }+\left| \left| \left| \omega \right| \right| \right| _{p\text {-var},[s,t]}\right) , \end{aligned}$$

which implies that $(y^n)$ is equicontinuous. Applying Proposition 5.28 of [8], we conclude that $y^n$ converges to some *y* along a subsequence in $\widehat{C}^{q}([s_0,s_1],\mathbb {R}^d)$. This proves the compactness of $\overline{F(B_1)}$. Hence, $F(B_1)$ is a relative compact set in $\widehat{C}^{q}([s_0,s_1],\mathbb {R}^d)$. We conclude that *F* is a compact operator from $B_1$ into itself. Therefore, by the Schauder-Tychonoff fixed point theorem (see e.g [25, Theorem 2.A, p. 56]), there exists a function $\hat{x}\in B_1$ such that $F(\hat{x})=\hat{x}$, thus there exists a solution $\hat{x}\in B_1$ of (3.1) on the interval $[s_0,s_1]$.

**Uniqueness of local solutions**.

Now, we assume that *x*, *y* are two solutions in $\widehat{C}^{q}([s_0,s_1],\mathbb {R}^d)$ of the Eq. (3.1) such that $x_{s_0}=y_{s_0}$. It follows that $F(x)=x$ and $F(y)=y$. Put

$$\begin{aligned} N_0 = \max \{\Vert x\Vert _{q\text {-var},[s_0,s_1]},\Vert y\Vert _{q\text {-var},[s_0,s_1]}\}, \end{aligned}$$

and $z=x-y$, we have $z_{s_0} = 0$ and

$$\begin{aligned} \Vert x\Vert _{\infty ,[s_0,s_1]},\Vert y\Vert _{\infty ,[s_0,s_1]}\le N_0. \end{aligned}$$

By virtue of Proposition 3.2(ii), we obtain

3.21 $$\begin{aligned} \left| \left| \left| z\right| \right| \right| _{q\text {-var},[s,t]}= & {} \left| \left| \left| x-y\right| \right| \right| _{q\text {-var},[s,t]}= \left| \left| \left| F(x)-F(y)\right| \right| \right| _{q\text {-var},[s,t]}\nonumber \\\le & {} M'_{N_0}(K+1) (1+ 2N_0^\delta ) \left( |z_s|+ \left| \left| \left| z\right| \right| \right| _{q\text {-var},[s,t]}\right) (t-s+\left| \left| \left| \omega \right| \right| \right| _{p\text {-var},[s,t]}). \end{aligned}$$

Applying Corollary 3.5 to the function *z*, since $z_{s_0} = 0$ we conclude that $\left| \left| \left| z\right| \right| \right| _{q\text {-var},[s_0,s_1]} =0$. That implies $z\equiv 0$ on $[s_0,s_1]$. The uniqueness of the local solution is proved.

**Step 2**: Next, by virtue of the additivity of the Riemann and Young integrals, the solution can be concatenated. Namely, let $0<t_1<t_2<t_3\le T$. Let $x_t$ be a solution of the Eq. (3.1) on $[t_1,t_2]$ and $y_t$ be a solution of the Eq. (3.1) on $[t_2,t_3]$ with $y(t_2)=x(t_2)$. Define a continuous function $z(\cdot ) : [t_1,t_3] \rightarrow \mathbb {R}^d$ by setting $z(t)=x(t)$ on $[t_1,t_2]$ and $z(t)=y(t)$ on $[t_2,t_3]$. Then $z(\cdot )$ is the solution of the Young differential equation (3.1) on $[t_1,t_3]$. Conversely, if $z_t$ is a solution on $[t_1,t_3]$ then its restrictions on $[t_1,t_2]$ and on $[t_2,t_3]$ are solutions of the corresponding equation with the corresponding initial values.

**Step 3**: Finally, apply the estimates (2.17) to the case of $\mu$ being defined by (3.19), we can easily get the unique global solution to the Eq. (3.1) on $[t_0,T]$.

Put $n_0= \min \{n: \tau _n> t_0 \}$. The interval $[t_0,T]$ can be covered by $N(T,\omega )-n_0+1$ intervals $[t_i,t_{i+1}]$, $i=\overline{0,N(T,\omega )-n_0+1}$, determined by times $t_i=\tau _{n_0+i-1}$, $i=1,\ldots , N(T,\omega )-n_0$, with parameter $\mu$ being defined by (3.19) and $t_{N(T,\omega )+1} :=T$. The arguments in **Step 1** are applicable to each of intervals $[t_i,t_{i+1}]$, $i=\overline{0,N(T,\omega )}$, implying the existence and uniqueness of solutions on those intervals. Then, starting at $x(t_0) = x_{t_0}$ the unique solution of (3.1) on $[t_0,t_1]$ is extended uniquely to $[t_1,t_2]$, then further by induction up to $[t_{N(T,\omega )-1},t_{N(T,\omega )}]$ and lastly to $[t_{N(T,\omega )},T]$. The solution *x* of (3.1) on $[t_0,T]$ then exists uniquely.

Furthermore, for all $\epsilon$ such that $q-\epsilon \ge p'$ the solution *x* belongs to $\widehat{C}^{q-\epsilon }([t_i,t_{i+1}],\mathbb {R}^d)$, for all $i=\overline{0,N(T,\omega )}$. Hence, $x\in \widehat{C}^{p'}([t_0,T],\mathbb {R}^d)$. $\square$

### Proposition 3.7

Assume that the conditions $\mathbf{H }_1-\mathbf{H }_3$ are satisfied. Let $0\le t_0<T$. Denote by $x(\cdot )=x(t_0,\cdot ,\omega ,x_{0})$ the solution of the Eq. (3.1) on $[t_0,T]$. Then there exist positive constants $C_1=C_1(T)$, $C_2=C_2(T)$ such that

3.22 $$\begin{aligned} \Vert x\Vert _{q\text {-}\mathrm{var},[t_0,T]}\le C_1[1{+}(T-t_0)^\alpha ] (1{+}|x_0|)(1{+}\left| \left| \left| \omega \right| \right| \right| _{p\text {-}\mathrm{var},[t_0,T]})e^{C_2\left| \left| \left| \omega \right| \right| \right| ^{p'}_{p\text {-}\mathrm{var},[t_0,T]}}, \end{aligned}$$

where $$p'=\max \{p,\frac{1}{\alpha }\}$$.

### Proof

Since *x* is a solution, $x=Fx$, hence by (3.11) we have

$$\begin{aligned} \left| \left| \left| x\right| \right| \right| _{q\text {-var},[s,t]}\le & {} M(K+2)\left( 1+\Vert x\Vert _{q\text {-var},[s,t]}\right) \left( (t-s)^{\alpha }+\left| \left| \left| \omega \right| \right| \right| _{p\text {-var},[s,t]} \right) \\\le & {} M(K+2)\left( (t-s)^{\alpha }+\left| \left| \left| \omega \right| \right| \right| _{p\text {-var},[s,t]} \right) \\&+\,M(K+2) (|x_s|+\left| \left| \left| x\right| \right| \right| _{q\text {-var},[s,t]})\left( (t-s)^{\alpha }+\left| \left| \left| \omega \right| \right| \right| _{p\text {-var},[s,t]} \right) . \end{aligned}$$

Use the arguments similar to that of the proof of Lemma 3.3 we conclude that there exist $C_1=C_1(T)$ and $C_2=C_2(T)$ such that (3.22) is satisfied. $\square$

In order to study the flow generated by the solution of system (3.1) in the next section, we need also to consider the backward version of (3.1) in the following form

3.23 $$\begin{aligned} x_t = x_T + \int _t^T f(s,x_s) ds + \int _t^T g(s,x_s) d\omega _s, \quad t\in [0,T], \end{aligned}$$

where $x_T\in \mathbb {R}^d$ is the initial value of the backward equation (3.23), the coefficient functions $f: [0,T] \times \mathbb {R}^d \rightarrow \mathbb {R}^d$, $g: [0,T] \times \mathbb {R}^d \rightarrow \mathbb {R}^d\times \mathbb {R}^m$ are continuous functions, and the driven force $\omega : [0,T] \rightarrow \mathbb {R}^m$ belongs to $\widehat{C}^{p}([0,T],\mathbb {R}^m)$.

### Theorem 3.8

*(Existence and uniqueness of solutions of backward equation)* Consider the backward equation (3.23) on [0, *T*]. Assume that the conditions $\mathbf{H }_1-\mathbf{H }_3$ hold. Then the backward equation (3.23) has a unique solution $x\in \widehat{C}^{q}([0,T],\mathbb {R}^d)$, where *q* is chosen as above satisfying (3.3).

### Proof

We make a change of variables

$$\begin{aligned} {\hat{f}}(u,x):= & {} f(T-u,x), \quad {\hat{g}}(u,x) := g(T-u,x),\quad {\hat{\omega }}(u) := \omega (T-u), \quad y_u := x_{T-u},\quad \\&u\in [0,T]. \end{aligned}$$

Then $x_T = y_0$, and by putting $v=T-t$ and $u=T-s$ we have

$$\begin{aligned} \int _t^T f(s,x_s) ds = \int _t^T f(T-u,x_{T-u}) ds = -\int _{T-t}^0 {\hat{f}}(u,y_u) du = \int _0^v {\hat{f}}(u,y_u) du. \end{aligned}$$

Furthermore, by virtue of the property (2.9) of the Young integral we have

$$\begin{aligned} \int _t^T g(s,x_s) d\omega _s = \int _t^T g(T-u,x_{T-u}) d\omega _{T-u} = \int _0^v {\hat{g}}(u,y_u) d{\hat{\omega }}_u. \end{aligned}$$

Therefore, the backward equation (3.23) is equivalent to the forward equation

3.24 $$\begin{aligned} y_v = y_0 + \int _0^v {\hat{f}}(u,y_u) du + \int _0^v {\hat{g}}(u,y_u) d{\hat{\omega }}_u, \quad v\in [0,T], \end{aligned}$$

where $y_0=x_T\in \mathbb {R}^d$. Now, we verify the conditions of Theorem 3.6 for the forward equation (3.24). First note that if $\omega \in \widehat{C}^{p}([0,T],\mathbb {R}^m)$ then ${\hat{\omega }}\in \widehat{C}^{p}([0,T],\mathbb {R}^m)$. Furthermore, the condition ($\mathbf{H }_1$) obviously holds for $\hat{g}$ and the condition (i) of ($\mathbf{H }_2$) holds for $\hat{f}$. For the condition (ii) of ($\mathbf{H }_2$) we note that if it holds for *f* then

$$\begin{aligned} |{\hat{f}}(v,x)| = |f(T-v,x)| \le a|x| + b(T-v) = a|x| + {\hat{b}}(v), \quad v\in [0,T], \end{aligned}$$

where ${\hat{b}}(v) = b(T-v) \in L^\frac{1}{1- \alpha }(0,T;\mathbb {R}^d)$ because ($\mathbf{H }_2$)(ii) is satisfied for *f*. Thus, ($\mathbf{H }_2$)(ii) is satisfied for $\hat{f}$. Consequently, Theorem 3.6 is applicable to the forward equation (3.24) implying that (3.24) has unique solution $y\in \widehat{C}^{q}([0,T],\mathbb {R}^d)$. Since (3.24) is equivalent to the backward equation (3.23) we have the theorem proved. $\square$

### Theorem 3.9

Suppose that the assumptions of Theorem 3.6 are satisfied. Denote by $X(t_0,\cdot ,\omega ,x_0)$ the solution of (3.1) starting from $x_0$ at time $t_0$, i.e. $X(t_0,t_0,\omega ,x_0)=x_0$. Then the solution mapping

$$\begin{aligned} X: [0,T]\times [0,T]\times \widehat{C}^{p}([0,T],\mathbb {R}^m)\times \mathbb {R}^d\rightarrow & {} \mathbb {R}^d,\\ (s,t,\omega ,z)\mapsto & {} X(s,t,\omega ,z), \end{aligned}$$

is continuous.

### Proof

First observe that, fixing $(\omega ,x_0) \in \widehat{C}^{p}([0,T],\mathbb {R}^m)\times \mathbb {R}^d$ and looking at forward and backward equations (3.1) and (3.23), we can extend the solution $X(t_0,\cdot ,\omega ,x_0)$ of (3.1), with the initial value $x_0$ at $t_0$ to the whole [0, *T*]. The proof is divided into several steps.

**Step 1 (Continuity w.r.t** $$x_0$$ **)**:

By Proposition 3.7, we can choose $N_0$ (depending on $x_0$, $\omega$) such that

$$\begin{aligned} \Vert X(t_0,\cdot ,\omega ',x'_0)\Vert _{q\text {-var},[0,T]}\le N_0 \end{aligned}$$

for all $t_0\in [0,T]$, $|x_0-x'_0|\le 1, \Vert \omega -\omega '\Vert _{p\text {-var},[0,T]}\le 1$. We use here, for short, notation $y_. = X(t_0,\cdot ,\omega ',x_0)$, $y'_. = X(t_0,\cdot ,\omega ',x'_0)$. Using arguments similar to that of the proof of Proposition 3.2(ii), we have

$$\begin{aligned}&|(y-y')_t-(y-y')_s|\\&\quad \le \int _s^t|f(u,y_u)-f(u,y'_u)|du+ \left| \int _s^t g(u,y_u)-g(u,y'_u)d\omega '_u\right| \\&\quad \le M_{N_0}^\prime (t-s)\Vert y-y'\Vert _{\infty ,[s,t]} \\&\qquad +\, M_{N_0}^\prime (K+1)\left| \left| \left| \omega '\right| \right| \right| _{p\text {-var},[s,t]}\left( |y_s-y'_s| + \left| \left| \left| y-y'\right| \right| \right| _{q\text {-var},[s,t]}\right) (2+2N_0^\delta )\\&\quad \le M_{N_0}^\prime (K+1)(2+2N_0^\delta ) \left( |y_s-y'_s| + \left| \left| \left| y-y'\right| \right| \right| _{q\text {-var},[s,t]}\right) \left( t-s+ \left| \left| \left| \omega '\right| \right| \right| _{p\text {-var},[s,t]}\right) . \end{aligned}$$

Due to Corollary 3.5, there exist constants $C_3,C_4$ depending on parameters of the equation (3.1) and $N_0$, such that

$$\begin{aligned} \left| \left| \left| y-y'\right| \right| \right| _{q\text {-var},[0,T]}\le |y_0-y'_0| C_3 e^{C_4\left| \left| \left| \omega '\right| \right| \right| _{p\text {-var},[0,T]}^p}\le |y_0-y'_0| C_3 e^{C_4(1+\Vert \omega \Vert _{p\text {-var},[0,T]})^p}. \end{aligned}$$

Therefore,

$$\begin{aligned} |x_t-y_t| \le |x_{t_0}-y_{t_0}| +\left| \left| \left| x-y\right| \right| \right| _{q\text {-var},[t_0,t]}\le |x_0-x'_0| \left( C_3 e^{C_4(1+\Vert \omega \Vert _{p\text {-var},[0,T]})^p} +1\right) . \end{aligned}$$

Consequently, we find a positive constants $C_1(T,\omega ,x_0)$ such that for all $t_0,t\in [0,T]$, all $\omega '$ such that $\Vert \omega '-\omega \Vert _{p\text {-var},[0,T]}<1$, we have

3.25 $$\begin{aligned} |X(t_0,t,\omega ',x_0')-X(t_0,t,\omega ',x_0)|\le C_1(T,\omega ,x_0)|x_0-x'_0|. \end{aligned}$$

**Step 2 (Continuity w.r.t.** $$\omega $$ **):**

Let $\omega '\in \widehat{C}^{p}([0,T],\mathbb {R}^m)$ be such that $\Vert \omega '-\omega \Vert _{p\text {-var},[0,T]}\le 1$. We use here, for short, notation $x_.=X(t_0,\cdot ,\omega , x_0)$, $x'_.=X(t_0,\cdot ,\omega ', x_0)$. For all $s<t$ in [0, *T*], we have

$$\begin{aligned}&|(x'-x)_t-(x'-x)_s|\\&\quad =\left| \int _s^t[f(u,x'_u)-f(u,x_u)]du+\int _s^t [g(u,x'_u)-g(u,x_u)]d\omega _u+\int _s^t g(u,x'_u)d(\omega '-\omega )_u\right| \\&\quad \le L_{N_0}(t-s)\Vert x'-x\Vert _{\infty ,[s,t]} +M(K+1)(1+\Vert x'\Vert _{q\text {-var},[s,t]})\left| \left| \left| \omega '-\omega \right| \right| \right| _{p\text {-var},[s,t]}\\&\qquad +\left| \left| \left| \omega '\right| \right| \right| _{p\text {-var},[s,t]}M_{N_0}'(K+1) \left( |(x'-x)_s|+\left| \left| \left| x'-x\right| \right| \right| _{q\text {-var},[s,t]}\right) \\&\qquad \times \left( 2+\left| \left| \left| x'\right| \right| \right| ^\delta _{q\text {-var},[0,T]}+\left| \left| \left| x\right| \right| \right| ^\delta _{q\text {-var},[0,T]}\right) \\&\quad \le C_5\left| \left| \left| \omega '-\omega \right| \right| \right| _{p\text {-var},[s,t]} + C_6\left( t-s+\left| \left| \left| \omega '\right| \right| \right| _{p\text {-var},[s,t]}\right) \left( |(x'-x)_s|+\left| \left| \left| x'-x\right| \right| \right| _{q\text {-var},[s,t]}\right) \\&\quad \le C_5\left| \left| \left| \omega '-\omega \right| \right| \right| _{p\text {-var},[s,t]} + C_6\left( t-s+\left| \left| \left| \omega '\right| \right| \right| _{p\text {-var},[s,t]}\right) \left( |(x'-x)_s|+\left| \left| \left| x'-x\right| \right| \right| _{q\text {-var},[s,t]}\right) , \end{aligned}$$

where $C_5,C_6$ depend on $N_0$. Consequently, by virtue of Lemma 2.3 we get

$$\begin{aligned} \left| \left| \left| x'-x\right| \right| \right| _{q\text {-var},[s,t]}\le & {} C_3\left| \left| \left| \omega '-\omega \right| \right| \right| _{p\text {-var},[s,t]} + C_4\left( t-s+\left| \left| \left| \omega \right| \right| \right| _{p\text {-var},[s,t]}\right) \\&\times \left( |(x'-x)_s|+\left| \left| \left| x'-x\right| \right| \right| _{q\text {-var},[s,t]}\right) . \end{aligned}$$

Now, since $x'_{t_0}-x_{t_0}=0$, using Collorary 3.5 on $[t_0,t]$ (or $[t,t_0]$ and use backward equation if $t<t_0$) we find positive constant $C_2(T,\omega ,x_0)$ such that

$$\begin{aligned} \left| \left| \left| x'-x\right| \right| \right| _{q\text {-var},[t_0,t]}\le C_2(T,\omega ,x_0)\left| \left| \left| \omega '-\omega \right| \right| \right| _{p\text {-var},[t_0,t]} \le C_2(T,\omega ,x_0)\Vert \omega '-\omega \Vert _{p\text {-var},[0,T]}. \end{aligned}$$

Therefore, for all $t_0,t\in [0,T]$,

3.26 $$\begin{aligned} | X(t_0,t,\omega ',x_0)-X(t_0,t,\omega ,x_0)|\le C_2(T,\omega ,x_0)\Vert \omega '-\omega \Vert _{p\text {-var},[0,T]}. \end{aligned}$$

**Step 3 (Continuity in all variables):**

Now we fix $(t_1,t_2,\omega ,x_0)$ and let $(t_1^\prime ,t_2^\prime ,\omega ^\prime ,x_0^\prime )$ be in a neighborhood of $(t_1,t_2,\omega ,x_0)$ such that

$$\begin{aligned} |t_1-t_1^\prime |, |t_2-t_2^\prime |, \Vert \omega -\omega ^\prime \Vert _{p\text {-var},[0,T]}, |x_0-x_0^\prime |\le 1. \end{aligned}$$

By triangle inequality and (3.25), (3.26), we have

$$\begin{aligned}&|X(t_1^\prime , t_2^\prime ,\omega ^\prime ,x_0^\prime ) - X(t_1,t_2,\omega ,x_0)|\\&\quad \le |X(t_1^\prime ,t_2^\prime ,\omega ^\prime ,x_0^\prime ) - X(t_1^\prime ,t_2^\prime ,\omega ',x_0)|+ |X(t_1^\prime ,t_2^\prime ,\omega ^\prime ,x_0) - X(t_1^\prime ,t_2^\prime ,\omega ,x_0)|\\&\qquad +\,|X(t_1^\prime ,t_2^\prime ,\omega ,x_0) - X(t_1,t_2',\omega ,x_0)|+ |X(t_1,t_2',\omega ,x_0)-X(t_1,t_2,\omega ,x_0)|\\&\quad \le (C_1(T,\omega ,x_0) + C_2(T,\omega ,x_0))(|x'_0-x_0|+\Vert \omega '-\omega \Vert _{p\text {-var},[0,T]})\\&\qquad +\, |X(t_1',t_2',\omega ,x_0)- X(t_1^\prime ,t_2^\prime ,\omega ,X(t_1,t_1',\omega ,x_0))| + \left| \left| \left| X(t_1,\cdot ,\omega ,x_0)\right| \right| \right| _{q\text {-var},[t_2,t_2']} \end{aligned}$$

It is obvious that when the triple $(|x'_0-x_0|,\Vert \omega '-\omega \Vert _{p\text {-var},[0,T]}, |t_2'-t_2|)$ tends to 0 we have $(C_1(T,\omega ,x_0) + C_2(T,\omega ,x_0))(|x-x_0|+\Vert \omega '-\omega \Vert _{p\text {-var},[0,T]})\rightarrow 0$ and $\left| \left| \left| X(t_1,.,\omega ,x_0)\right| \right| \right| _{q\text {-var},[t_2,t_2']}\rightarrow 0$. As for the remaining term, let $|t_1'-t_1|$ be small enough so that $|X(t_1,t_1',\omega ,x_0)-x_0|\le 1$, using (3.25) again we obtain

$$\begin{aligned}&|X(t_1',t_2',\omega ,X(t_1,t_1',\omega ,x_0))- X(t_1^\prime ,t_2^\prime ,\omega ,x_0)|\\&\quad \le C_1(T,\omega ,x_0) |X(t_1,t_1',\omega ,x_0))-x_0|\\&\quad \le C_1(T,\omega ,x_0)\left| \left| \left| X(t_1,\cdot , \omega ,x_0)\right| \right| \right| _{q\text {-var},[t_1,t_1']}, \end{aligned}$$

hence $|X(t_1',t_2',\omega ,X(t_1,t_1',\omega ,x_0))- X(t_1^\prime ,t_2^\prime ,\omega ,x_0)| \rightarrow 0$ as $|t_1'-t_1|\rightarrow 0$. Summing up the above arguments, we conclude that *X* is continuous. $\square$

### Remark 3.10

The time interval in Theorems 3.6 to  3.9 needs not be [0, *T*]. It can be $[t_0,t_0+T]$ for any $t_0\in \mathbb {R}$, $T>0$.

## Topological Flow Generated by Young Differential Equations

In this section we show that the solution of a nonautonomous Young differential equation generates a two-parameter flow on the phase space $\mathbb {R}^d$, thus we can study the long term behavior of the solution flow using the tools of the theory of dynamical systems. We also discuss the autonomous situation, in which we show that the solution then satisfies the cocycle property, thus generates a topological skew product flow. The reader is referred to the work [15] and [17, 18] for the smoothness and diffeomorphism property of the flow.

For simplicity of the presentation, we will assume from now on that for any given $T>0$ all hypotheses $\mathbf{H }_1-\mathbf{H }_3$ hold on [0, *T*].

### Topological Two-Parameter Flows for Nonautonomous Systems

#### Theorem 4.1

(Different trajectories do not intersect) Assume that the conditions $\mathbf{H }_1-\mathbf{H }_3$ hold. Let $x_t$ and $\hat{x}_t$ be two solutions of the Young differential equation (3.1) on [0, *T*]. If $x_a = \hat{x}_a$ for some $a\in [0,T]$ then $x_t =\hat{x}_t$ for all $t\in [0,T]$. In other words, two solutions of the differential equation (3.1) either coincide or do not intersect.

#### Proof

Suppose that $x_a=\hat{x}_a$ for some $a\in [0,T]$. If $a=0$ then by the uniqueness of the solution provided by Theorem 3.6, $x_t =\hat{x}_t$ for all $t\in [0,T]$. Let $a\in (0,T]$. Since the restrictions of the functions $x_t$ and $\hat{x}_t$ on [*a*, *T*] are solutions of the Eq. (3.1) starting from *a*, Theorem 3.6 implies that $x_t=\hat{x}_t$ for all $t\in [a,T]$. Now, consider the restrictions of the functions $x_t$ and $\hat{x}_t$ on [0, *a*]. They are solutions of the equations

$$\begin{aligned} x_t = x_0 + \int _0^t f(s,x_s) ds + \ \int _0^t g(s,x_s) d\omega _s, \quad t\in [0,a], \end{aligned}$$

with the initial values $x_0$ and ${\hat{x}}_0$ respectively. Since $x_a=\hat{x}_a$, the two functions $x_t$ and $\hat{x}_t$ are solutions of the same backward equation

4.1 $$\begin{aligned} x_t = x_a - \int _t^a f(s,x_s) ds - \int _t^a g(s,x_s) d\omega _s, \quad t\in [0,a], \end{aligned}$$

with the same initial value $x_a$. Theorem 3.8 asserts the uniqueness of solution of (4.1) on [0, *a*], hence $x_t$ must coincide with $\hat{x}_t$ on [0, *a*] and the theorem is proved.$\square$

#### Remark 4.2

(Locality of Young differential equations) By virtue of Theorems 3.6, 3.8 and 4.1, under the assumptions of Theorem 3.6, the Eq. (3.1) has locality properties like ODE: we can solve it locally and extend the solution both forward and backward, and any two solutions meeting each other at some time should coincide in the common interval of definitions.

Now, in analog with the theory of ordinary differential equation we give a definition of the Cauchy operator of the Eq. (1.1), which is an operator in $\mathbb {R}^d$ acting along trajectoties of (1.1).

#### Definition 4.3

*(Cauchy operator)* Suppose that on any compact interval of $\mathbb {R}$ the conditions $\mathbf{H }_1-\mathbf{H }_3$ hold. For any $-\infty< t_1\le t_2<+\infty$, any $\omega \in \widetilde{C}^p(\mathbb {R},\mathbb {R}^m)$ the *Cauchy operator* $X(t_1,t_2,\omega ,\cdot )$ of the Eq. (1.1) is defined as follows:

$$\begin{aligned} X(t_1,t_2,\omega ,\cdot ) : \mathbb {R}^d \rightarrow \mathbb {R}^d \end{aligned}$$

is the mapping along trajectories of (1.1) from time moment $t_1$ to time moment $t_2$, i.e., for any vector $x_{t_1}\in \mathbb {R}^d$ we define $X(t_1,t_2,\omega ,x_{t_1})$ to be the vector $x_{t_2}\in \mathbb {R}^d$ which is the value of the solution *x* of the equation

$$\begin{aligned} x_t = x_{t_1} + \int _{t_1}^t f(s,x_s) ds + \int _{t_1}^t g(s,x_s) d\omega _s, \quad t\in [t_1,t_2], \end{aligned}$$

evaluated at time $t_2$.

#### Theorem 4.4

Assume that the conditions $\mathbf{H }_1-\mathbf{H }_3$ hold on any compact interval of $\mathbb {R}$. For any $-\infty< t_1\le t_2<+\infty$ the Cauchy operator $X(t_1,t_2,\omega ,\cdot )$ of (1.1) is a homeomorphism. Moreover, $X(t_1,t_1,\omega ,\cdot )=id$.

#### Proof

By Theorem 4.1 the Cauchy operator $X(t_1,t_2,\omega ,\cdot )$ is an injection. Using arguments of the proof of Theorem 4.1 we get that the equation

4.2 $$\begin{aligned} x_t = x_{t_1} + \int _{t_1}^t f(s,x_s) ds + \int _{t_1}^t g(s,x_s) d\omega _s, \quad t\in [t_1,t_2], \end{aligned}$$

with the terminal value $x_{t_2}\in \mathbb {R}^d$ and unknown initial value $x_{t_1}$, is equivalent to the following initial value problem for the backward equation on $[t_1,t_2]$

4.3 $$\begin{aligned} x_t = x_{t_2} - \int _t^{t_2} f(s,x_s) ds - \int _t^{t_2} g(s,x_s) d\omega _s, \quad t\in [t_1,t_2], \end{aligned}$$

with initial value $x_{t_2}\in \mathbb {R}^d$, hence Theorem 3.8 is applicable and provides existence of solution for any terminal value $x_{t_2}$ of the forward equation on $[t_1,t_2]$. Consequently, the Cauchy operator $X(t_1,t_2,\omega ,\cdot )$ is a surjection, thus a bijection.

It is clear from the proof of Theorems 3.6 and 3.9 that the solutions of (1.1) depend continuously on the initial values. Therefore, the Cauchy operator $X(t_1,t_2,\omega ,\cdot )$ acts continuously on $\mathbb {R}^d$. Similar conclusion holds for the inverse $X^{-1}(t_1,t_2,\omega ,\cdot )$ by using backward equation. Hence $X(t_1,t_2,\omega ,\cdot )$ is a homeomorphism and trivially $X(t_1,t_1,\omega ,\cdot )=id$. $\square$

Following [13, p. 114], below we introduce the concept of two parameter flows.

#### Definition 4.5

*(Two-parameter flow)* A family of mappings $X_{s,t} : \mathbb {R}^d \rightarrow \mathbb {R}^d$ depending on two real variables $s,t\in [a,b] \subset \mathbb {R}$ is call a *two-parameter flow of homeomorphisms of* $\mathbb {R}^d$ *on* [*a*, *b*] if it satisfies the following conditions:

(i) For any $s,t\in [a,b]$ the mapping $X_{s,t}$ is a homeomorphism of $\mathbb {R}^d$;
(ii) $X_{s,s} = id$ for any $s\in [a,b]$;
(iii) $X_{s,t}^{-1} = X_{t,s}$ for any $s,t\in [a,b]$;
(iv) $X_{s,t} = X_{u,t}\circ X_{s,u}$ for any $s,t,u\in [a,b]$.

#### Theorem 4.6

*(Two-parameter flow generated by Young differential equations)* Assume that the conditions $\mathbf{H }_1-\mathbf{H }_3$ hold on any compact interval of $\mathbb {R}$. The family of Cauchy operators of (1.1) generates a two parameter flow of homeomorphisms of $\mathbb {R}^d$. Namely, for $-\infty< t_1\le t_2<+\infty$ and $\omega \in \widetilde{C}^p(\mathbb {R},\mathbb {R}^m)$ we define $X(t_1,t_2,\omega ,\cdot )$ according to Definition 4.3 and setting $X(t_2,t_1,\omega ,\cdot ) := X^{-1}(t_1,t_2,\omega ,\cdot )$, then the family $X(t_1,t_2,\omega ,\cdot )$, $t_1,t_2\in [0,T]$, is a two parameter flow of homeomorphisms of $\mathbb {R}^d$ on [0, *T*]. Furthermore, the flow is continuous.

#### Proof

Conditions (i)–(ii) of Definition 4.5 follow from Theorem 4.4.

Condition (iii) of Definition 4.5 follows from the definition $X(t_2,t_1,\omega ,\cdot ) := X^{-1}(t_1,t_2,\omega ,\cdot )$ for $t_1\le t_2$. Actually, it is seen from the proof of Theorem 4.4 that the inverse $X(t_2,t_1,\omega ,\cdot )$ satisfies the backward equation (4.3).

Condition (iv) of Definition 4.5 follows from the definition of the Cauchy operators and Theorem  4.1.

The continuity of the flow follows directly from Theorem 3.9. $\square$

### Topological Skew Product Flows for Autonomous Systems

In this subsection we restrict the discussion to the autonomous system

4.4 $$\begin{aligned} dx_t = f(x_t)dt + g(x_t)d\omega _t \end{aligned}$$

where *f*, *g* are time independent and sastisfy the assumptions $\mathbf{H }_1-\mathbf{H }_3$. We consider $\omega$ in the space $\widetilde{C}^{p}_0(\mathbb {R},\mathbb {R}^m) := \{ \omega \in \widetilde{C}^{p}_0(\mathbb {R},\mathbb {R}^m), \omega (0) = 0\}$ and introduce the shift operator $\theta : \mathbb {R}\times \widetilde{C}^{p}_0(\mathbb {R},\mathbb {R}^m) \rightarrow \widetilde{C}^{p}_0(\mathbb {R},\mathbb {R}^m)$ by

$$\begin{aligned} \theta _t \omega (\cdot ) := \omega (t+\cdot ) - \omega (t). \end{aligned}$$

It is easy to check that $\theta _{t+s}\omega = \theta _t \circ \theta _s \omega$ for all $t,s\in \mathbb {R}$ and $\omega \in \widetilde{C}^{p}_0(\mathbb {R},\mathbb {R}^m)$. Moreover, it is followed from [3, Theorem 5] that $\theta$ is continuous w.r.t. $(t,\omega )$, thus $(\widetilde{C}^{p}_0(\mathbb {R},\mathbb {R}^m),d,\theta )$ is a continuous dynamical system. On the other hand, it follows from definition of Young integral that

$$\begin{aligned} \int _{s+\tau }^{t+\tau } y_u d\omega (u)=\int _s^t y_{u+\tau } d\theta _\tau \omega (u),\ \forall s, t, \tau \in \mathbb {R}\end{aligned}$$

(see [5] for a version using fractional derivatives). Hence from the existence and uniqueness theorem, the solution $X(t,s,\omega ,x_0)$ of the Young equation (4.4) satisfies

$$\begin{aligned} X(t,s,\omega ,x_0) = X(t-s,0,\theta _s \omega ,x_0),\ \forall t,s \in \mathbb {R}, \end{aligned}$$

therefore the mapping $\varphi : \mathbb {R}\times \widetilde{C}^{p}_0(\mathbb {R},\mathbb {R}^m) \times \mathbb {R}^d \rightarrow \mathbb {R}^d$ defined by $\varphi (t,\omega )x_0 := X(t,0,\omega ,x_0)$ possesses a cocycle property

$$\begin{aligned} \varphi (t+s,\omega )x_0= \varphi (t,\theta _s \omega )\circ \varphi (s,\omega )x_0,\ \forall x_0\in \mathbb {R}^d, \omega \in \widetilde{C}^{p}_0 (\mathbb {R},\mathbb {R}^m), t,s \in \mathbb {R}. \end{aligned}$$

The problem of generation of the random dynamical systems [1] from stochastic differential equations driven by fractional Brownian noise has been discussed in [3, 5, 9], to name a few, where they solve the stochastic equation in the path-wise sense as in (4.4) for each realization $\omega$ of the fractional Brownian motion. Here in our deterministic setting, due to the fact that the shift dynamical system $\theta$ and the solution Cauchy operator are continuous, it follows that the skew product flow defined by

4.5 $$\begin{aligned} \Theta : \mathbb {R}\times \widetilde{C}^{p}_0(\mathbb {R},\mathbb {R}^m) \times \mathbb {R}^d\rightarrow & {} \widetilde{C}^{p}_0(\mathbb {R},\mathbb {R}^m)\times \mathbb {R}^d \nonumber \\ \Theta _t (\omega ,x_0):= & {} (\theta _t \omega , X(t,\omega ,x_0)) \end{aligned}$$

is a continuous mapping which satisfies the group property, i.e. $\Theta _{t+s} = \Theta _t \circ \Theta _s$, for all $t,s \in \mathbb {R}$. Therefore it is a topological skew-product dynamical system.
